# Isolation and characterization of gluten protein types from wheat, rye, barley and oats for use as reference materials

**DOI:** 10.1371/journal.pone.0172819

**Published:** 2017-02-24

**Authors:** Kathrin Schalk, Barbara Lexhaller, Peter Koehler, Katharina Anne Scherf

**Affiliations:** Deutsche Forschungsanstalt für Lebensmittelchemie, Leibniz Institut, Freising, Germany; Tulane University, UNITED STATES

## Abstract

Gluten proteins from wheat, rye, barley and, in rare cases, oats, are responsible for triggering hypersensitivity reactions such as celiac disease, non-celiac gluten sensitivity and wheat allergy. Well-defined reference materials (RM) are essential for clinical studies, diagnostics, elucidation of disease mechanisms and food analyses to ensure the safety of gluten-free foods. Various RM are currently used, but a thorough characterization of the gluten source, content and composition is often missing. However, this characterization is essential due to the complexity and heterogeneity of gluten to avoid ambiguous results caused by differences in the RM used. A comprehensive strategy to isolate gluten protein fractions and gluten protein types (GPT) from wheat, rye, barley and oat flours was developed to obtain well-defined RM for clinical assays and gluten-free compliance testing. All isolated GPT (ω5-gliadins, ω1,2-gliadins, α-gliadins, γ-gliadins and high- and low-molecular-weight glutenin subunits from wheat, ω-secalins, γ-75k-secalins, γ-40k-secalins and high-molecular-weight secalins from rye, C-hordeins, γ-hordeins, B-hordeins and D-hordeins from barley and avenins from oats) were fully characterized using analytical reversed-phase high-performance liquid chromatography (RP-HPLC), sodium dodecyl sulfate polyacrylamide gel electrophoresis (SDS-PAGE), N-terminal sequencing, electrospray-ionization quadrupole time-of-flight mass spectrometry (LC-ESI-QTOF-MS) and untargeted LC-MS/MS of chymotryptic hydrolyzates of the single GPT. Taken together, the analytical methods confirmed that all GPT were reproducibly isolated in high purity from the flours and were suitable to be used as RM, e.g., for calibration of LC-MS/MS methods or enzyme-linked immunosorbent assays (ELISAs).

## Introduction

Wheat is the third most important cereal in terms of production worldwide (729 × 10^6^ t in 2014) [1], but the consumption of wheat and closely related cereals (rye, barley and, in rare cases, oats) may be harmful to predisposed individuals, because specific proteins are responsible for triggering hypersensitivities such as wheat allergy, celiac disease (CD) and non-celiac gluten sensitivity (NCGS) [2–4]. The major causative agents are the storage proteins (gluten) of the aforementioned grains, but other proteins such as lipid-transfer-proteins, puroindolines and amylase-trypsin-inhibitors (ATIs) also have the potential to cause harmful effects [5,6]. Cereal grains contain hundreds of different protein components which are traditionally classified into four so-called Osborne fractions: albumins soluble in water, globulins soluble in salt solution, prolamins soluble in aqueous alcohol and insoluble glutelins, which are only alcohol-soluble in the presence of reducing agents. Albumins and globulins (ALGL, ≈ 20–25% of grain proteins) mainly comprise metabolic and protective proteins such as enzymes and enzyme inhibitors whereas prolamins and glutelins (≈ 75–80% of grain proteins) serve as storage proteins. The common names of these closely related gluten proteins are gliadins (prolamins) and glutenins (glutelins) of wheat, secalins of rye, hordeins of barley and avenins of oats. Based on homologous amino acid sequences and similar molecular weights (M_r_), the gluten proteins can be divided into the high-molecular-weight (HMW), the medium-molecular-weight (MMW) and the low-molecular-weight (LMW) group [7]. Each group contains numerous related gluten protein types (GPT) with different numbers of single proteins within each type, e.g., HMW-glutenin subunits (GS) with 3–5 proteins and α-gliadins and LMW-GS with more than 20 proteins [8]. Modifications of amino acid sequences caused by nucleotide insertion, deletion or exchange are responsible for the heterogeneity within each type.

Numerous research papers have been published concerning identification and characterization of proteins that trigger wheat hypersensitivities [9–11]. Well-defined proteins are essential for clinical studies [12,13], diagnostic purposes and as reference materials (RM) for food analysis [14], such as the Prolamin Working Group (PWG)-gliadin [15]. Different RM have been used in these papers, but a thorough characterization of the protein source, content and composition often is either missing or proprietary material is used. Gluten and gliadin preparations frequently used for both clinical and analytical purposes were shown to be strongly different in protein content and proportions of ALGL, prolamin and glutelin fractions [16]. Considering the additional lack of reproducible RM production, the quality of assays for diagnosis and food analysis is variable and may lead to questionable and contradictory conclusions. Defined single recombinant proteins were applied in a few cases, e.g., a panel of 11 α-gliadins for CD-specific T-cell proliferation assays [17,18], γ1-gliadin for CD diagnosis [19], HMW-GS 1Dy10 for the investigation of CD serology [20] or HMW-GS 1Ax2 and ω5-gliadin for WDEIA diagnosis [11,21]. However, a single recombinant protein may not be representative for the corresponding GPT, because each GPT consists of several proteins. Using the complete protein mixture isolated from the natural source may therefore improve the accuracy of clinical and food analytical assays.

The aim of the present study was to develop and apply a comprehensive strategy to isolate well-defined gluten protein fractions and GPT from wheat, rye, barley and oat flours suitable as RM for clinical assays and gluten-free compliance testing, e.g., by enzyme-linked immunosorbent assays (ELISAs) or liquid chromatography-mass spectrometry (LC-MS). All isolated GPT were extensively characterized using analytical reversed-phase high-performance liquid chromatography (RP-HPLC), sodium dodecyl sulfate polyacrylamide gel electrophoresis (SDS-PAGE), N-terminal sequencing, electrospray-ionization quadrupole time-of-flight MS (LC-ESI-QTOF-MS) and untargeted LC-MS/MS of chymotryptic hydrolyzates of the single GPT.

## Material and methods

### Chemicals and flours

All chemicals and solvents were at least pro analysi or HPLC grade. Water for HPLC was purified using a Milli-Q Gradient A10 system (Millipore, Schwalbach, Germany). PWG-gliadin [15] used for calibration was provided by Prof. Dr. Peter Koehler, chairman of the PWG. Grains of four cultivars (cv.) each of wheat (cv. Akteur, I.G. Pflanzenzucht, Munich, Germany; cv. Julius, KWS Lochow, Bergen, Germany; cv. Pamier, Lantmännen SW Seed, JK Bergen op Zoom, The Netherlands; cv. Tommi, Nordsaat Saatzucht, Langenstein, Germany), rye (cv. Brasetto, cv. Conduct, cv. Palazzo, cv. Visello, KWS Lochow), barley (cv. Grace, cv. Marthe, Nordsaat Saatzucht; cv. Lomerit, KWS Lochow; cv. Sandra, I.G. Pflanzenzucht) and oats (cv. Aragon, cv. Ivory, cv. Scorpion, Nordsaat Saatzucht; cv. Flämingsgold, KWS Lochow), all harvested in 2013, were mixed in a 1+1+1+1 mass ratio and shaken overhead (Turbula, Willy A. Bachofen Maschinenfabrik, Muttenz, Switzerland) for 24 h to obtain homogeneous grain mixtures. The mixed wheat, rye and barley grains were milled into white flour using a Quadrumat Junior Mill (Brabender, Duisburg, Germany) followed by sieving (mesh size 200 μm). Oat grains were milled with a laboratory grinder (A10, IKA-Werke, Staufen, Germany) and sifted.

### Analytical characterization of the flours

The moisture and ash contents were determined according to International Association for Cereal Science and Technology (ICC) Standards 110/1 [22] and 104/1 [23]. The nitrogen contents were determined by the Dumas combustion method using a TruSpec nitrogen analyzer (Leco, Moenchengladbach, Germany) and converted to crude protein (CP) contents by multiplying with a factor of 5.7 according to ICC Standard 167 [24]. The quantities of ALGL, prolamin and glutelin fractions as well as GPT were determined according to the modified Osborne procedure [25,26]. The flours (100 mg) were extracted sequentially with (a) salt solution (2 × 1.0 mL; 0.4 mol/L NaCl with 0.067 mol/L Na_2_HPO_4_/KH_2_PO_4_, pH 7.6) for 10 min at 22°C (ALGL); (b) with ethanol/water (60/40, v/v) (3 × 0.5 mL) for 10 min at 22°C (prolamins); and (c) glutelin solution (2 × 1.0 mL; 2-propanol/water (50/50, v/v)/0.1 mol/l Tris-HCl, pH 7.5, containing 2 mol/L (w/v) urea and 0.06 mol/L (w/v) dithiothreitol (DTT)) for 30 min at 60°C under nitrogen (glutelins). The suspensions were centrifuged (3750 × *g*, 20 min, 22°C), the corresponding supernatants combined, made up to 2.0 mL with the respective extraction solvent and filtered (0.45 μm). Aliquots of the rye and barley prolamin fractions, respectively, were additionally analyzed after reduction (addition of 1% (w/v) DTT, 60°C, 30 min) [27]. All fractions were analyzed by analytical RP-HPLC [28]: instrument: Jasco XLC (Jasco, Gross-Umstadt, Germany); column: Acclaim^TM^ 300 C_18_ (3 μm, 30 nm, 2.1 × 150 mm, Thermo Fisher Scientific, Braunschweig, Germany); temperature: 60°C; injection volume: 20 μL of ALGL and glutelin extracts; 10 μL of prolamin extracts; elution solvents: (A) water/trifluoroacetic acid (TFA) (999/1, v/v), (B) acetonitrile/TFA (999/1, v/v); gradient for ALGL: 0 min 0% B, 0.5 min 20% B, 7 min 60% B, 7.1–11 min 90% B, 11.1–17 min 0% B; gradient for prolamins and glutelins: 0 min 0% B, 0.5 min 24% B, 20 min 56% B, 20.1–24.1 min 90% B, 24.2–30 min 0% B; flow rate: 0.2 mL/min; detection: UV absorbance at 210 nm; software: Chrompass (Jasco). PWG-gliadin [15] dissolved in ethanol/water was used for external calibration in the range of 11.6 to 46.6 μg to calculate the protein contents of the ALGL, prolamin and glutelin fractions. The amounts of GPT were calculated from the absorbance area of each GPT relative to the total absorbance area of the respective prolamin or glutelin fraction. All determinations were done in triplicates.

### Defatting of the flours

100 g of flour each were stirred three times at 22°C for 30 min with 250 mL *n*-pentane/ethanol (95/5, v/v) followed by stirring once with 250 mL *n*-pentane [29]. The suspensions were centrifuged (3750 × *g*, 15 min, 22°C) and the solvent discarded. After the last extraction step the defatted flour residue was vacuum-dried overnight on a filter sheet and homogenized carefully.

### Preparation of gluten protein fractions

Defatted flours (2 × 50 g) were extracted three times each with 200 mL of (a) salt solution by homogenizing with an Ultra Turrax blender (16 000 rpm, IKA-Werke, Staufen, Germany) in a centrifuge vessel for 5 min at 22°C. The suspensions were centrifuged (3750 × g, 25 min, 22°C) and the supernatants discarded (→ ALGL fraction). The sediments were extracted three times with 200 mL of (b) ethanol/water as described for the ALGL fraction. The resulting supernatants were combined, concentrated under reduced pressure, dialyzed (M_r_ cut-off: 12 000–14 000, Medicell Membranes, London, UK) and lyophilized (→ prolamin fraction). Then, the sediments were extracted three times under nitrogen with 200 mL of (c) glutelin solution (see above) by homogenizing with an Ultra Turrax blender for 5 min, stirring for 30 min at 60°C, cooling and centrifugation as described. The supernatants were combined, concentrated, dialyzed and lyophilized (→ glutelin fraction). For oat flour, the extraction was stopped after the prolamin fraction (M_r_ cut-off for dialysis: 7 000, Medicell Membranes), because oat glutelins are mainly composed of polymeric 12S globulins [30]. The CP contents of the dried prolamin and glutelin fractions were determined according to ICC Standard 167 (n = 3) [24].

### Preparation of gluten protein types

**Fig 1:**
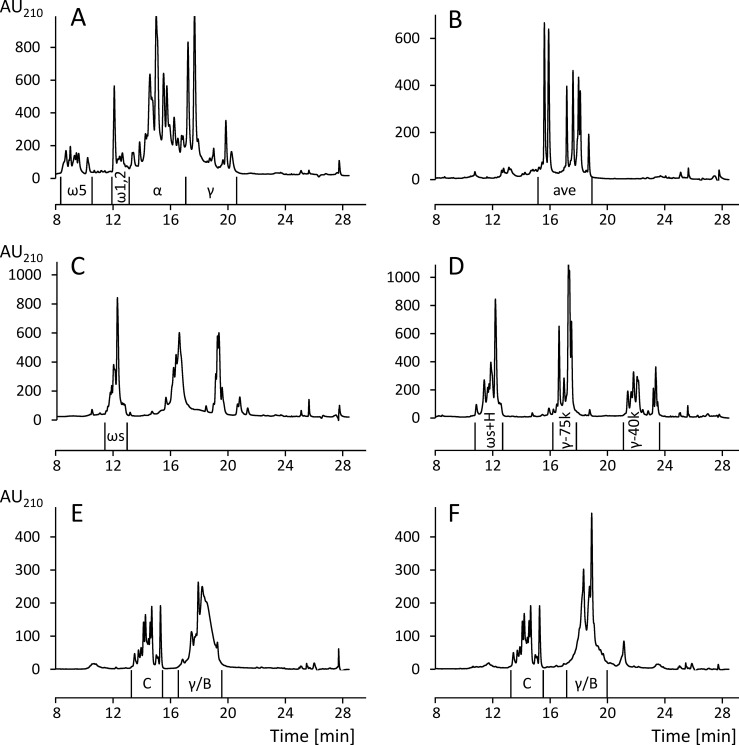
RP-HPLC chromatograms of the prolamin fractions. (A) Wheat prolamins, (B) oat prolamins, (C) rye prolamins, unreduced, (D) rye prolamins, reduced with 1% (w/v) DTT, (E) barley prolamins, unreduced, (F) barley prolamins, reduced with 1% (w/v) DTT. AU, absorbance units at 210 nm, ω5, ω5-gliadins, ω1,2, ω1,2-gliadins, α, α-gliadins, γ, γ-gliadins, ave, avenins, ωs, ω-secalins, ωs+H, ω- and high-molecular-weight (HMW)-secalins, γ-75k, γ-75k-secalins, γ-40k, γ-40k-secalins, C, C-hordeins, γ/B, γ-hordeins and B-hordeins.

**Fig 2:**
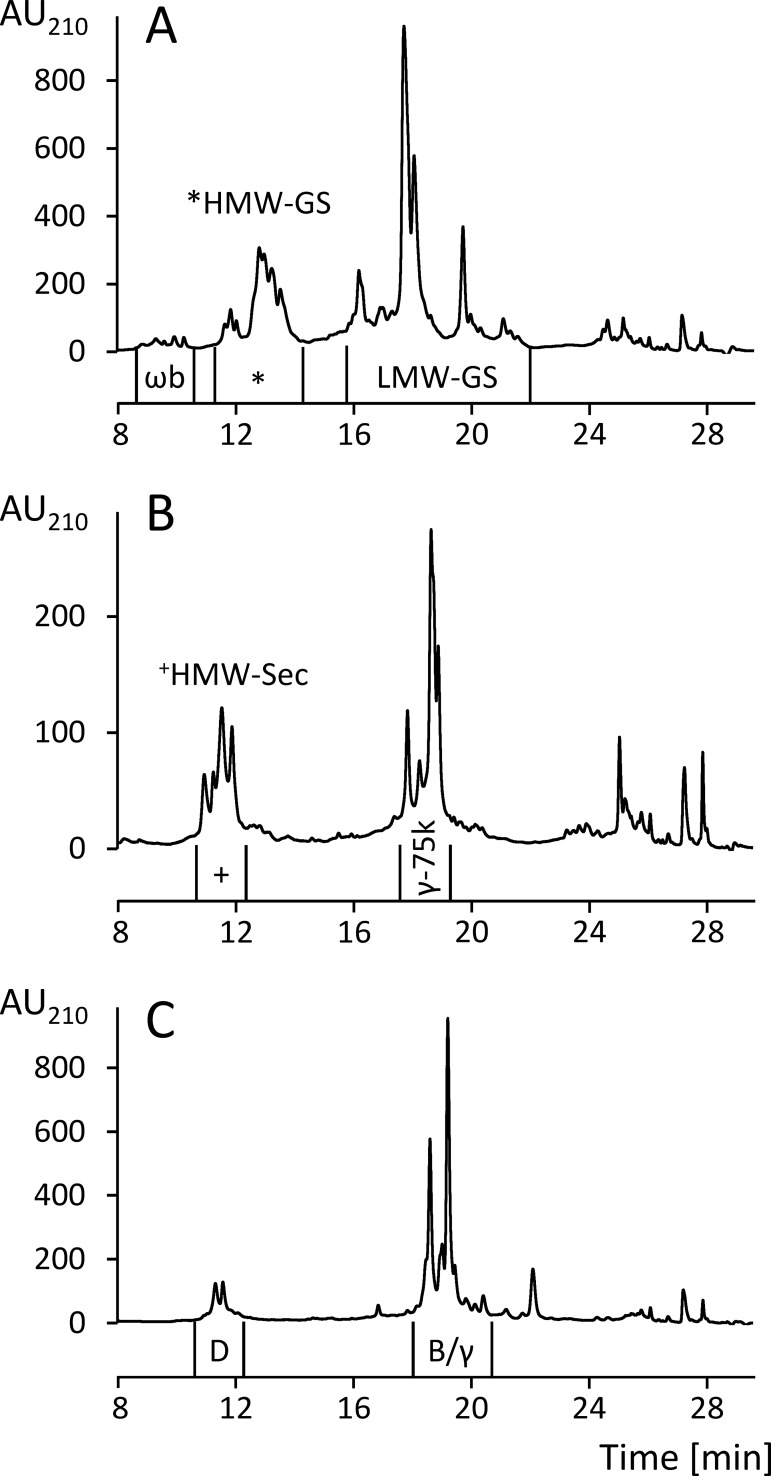
RP-HPLC chromatograms of the glutelin fractions. (A) Wheat glutelins, (B) rye glutelins, (C) barley glutelins, all reduced with 1% (w/v) DTT. AU, absorbance units at 210 nm, ωb, ωb-gliadins, HMW-GS, high-molecular-weight glutenin subunits, LMW-GS, low-molecular-weight glutenin subunits, HMW-Sec, HMW-secalins, γ-75k, γ-75k-secalins, γ-40k, γ-40k-secalins, D, D-hordeins, B/γ, B-hordeins and γ-hordeins.

The wheat, rye and barley prolamin fractions (100 mg) were dissolved in 10 mL of ethanol/water. The rye prolamin and the wheat, rye and barley glutelin fractions (100 mg) were dissolved in 10 mL of glutelin solution. All solutions were filtered (0.45 μm) and the following conditions were set for the preparative RP-HPLC method: pump: PU-2087 Plus (Jasco); autosampler: AS-2055 Plus (Jasco); column: Jupiter C_18_ (5 μm, 30 nm, 10 × 250 mm, Phenomenex, Aschaffenburg, Germany); temperature: 50°C; injection volume: 400 μL of prolamins, 700 μL of glutelins; elution solvent: (A) water/TFA (999/1, v/v), (B) acetonitrile/TFA (999/1, v/v); gradient: 0–2 min 0% B, 4 min 24% B, 52 min 56% B, 53 – 58 min 90% B, 65–69 min 0% B; flow rate: 2.0 mL/min; UV detector: UV-2075 Plus (Jasco); detection: UV absorbance at 210 nm; fraction collector: CHF-122SC (Advantec MFS, Dublin, CA, USA); software: Galaxie chromatography data system, version 1.10.0.5590 (Jasco). The GPT were separated according to their characteristic retention times (Figs 1 and 2), collected from several runs, pooled, concentrated under reduced pressure and lyophilized. Oat prolamins (avenins) were not further fractionated.

### Characterization of gluten protein types

#### Protein content

Due to the availability of only small amounts (mg) of GPT, 1 mg of the lyophilized GPT were dissolved in 1 mL of ethanol/water (GPT isolated from prolamin fractions) or glutelin solution (GPT isolated from glutelin fractions), filtered (0.45 μm), injected (20 μL) into the analytical RP-HPLC system (prolamin and glutelin gradient) and the protein concentrations were calculated from external calibration with PWG-gliadin (2.9–46.6 μg) as described above. This re-chromatography also allowed verifying the purity and identity of the isolated GPT by comparing their retention times with those determined previously during the analyses of the corresponding prolamin and glutelin fractions.

#### SDS-PAGE

SDS-PAGE was carried out according to Lagrain et al. [31] using a homogeneous NuPAGE 10% polyacrylamide - Bis-Tris gel (Invitrogen, Carlsbad, CA, USA) and a MOPS-Tris running buffer (pH 7.7) containing DTT (5 mmol/L) added to the inside chamber. The isolated protein fractions or GPT (1.5 mg) were dissolved in 1 mL of extraction buffer under reducing conditions (DTT, 50 mmol/L), incubated for 24 h, heated to 60°C for 10 min while shaking and centrifuged (5000 × *g*, 5 min, 22°C). Per sample, 2–5 μL were applied to the slots. A mixture of seven proteins (M_r_ 6 500–200 000) was used as marker. The running time was 40 min at 200 V and 115 mA. After the run, the proteins were fixed for 30 min in 12% trichloroacetic acid, stained for 30 min with Coomassie Brilliant Blue R-250 and destained twice [31]. The gels were scanned, the images converted to grayscale, the lanes of interest plotted as x/y-diagrams and the peaks integrated using ImageJ open source software (National Institute of Mental Health, Bethesda, MD, USA) [32].

#### N-terminal sequence analysis

Isolated GPT were dissolved in acetonitrile/water (30/70, v/v) containing 0.1% (v/v) TFA. The amount of protein applied onto the polyvinylidene difluoride membrane was between 50 and 100 pmol. Sequencing was carried out by automated Edman degradation on a protein sequencer Procise 492 (Applied Biosystems, Carlsbad, CA, USA) running in the pulsed-liquid mode with ten degradation cycles [33].

#### LC-ESI-QTOF-MS

An ESI-QTOF-MS (microTOF-Q, Bruker Daltonics, Bremen, Germany) coupled with an UltiMate 3000 HPLC system (Dionex, Idstein, Germany) was used for LC-MS experiments [34]. The stationary phase was an XBridge Protein BEH C_4_ column (3.5 μm, 30 nm, 2.1 × 150 mm, Waters, Milford, MA, USA). The mobile phase was (A) water/TFA (999/1, v/v) and (B) acetonitrile/TFA (999/1, v/v) with a linear elution gradient from 0–0.4 min 0% B, 0.5 min 24% B, 20 min 56% B, 20.1–32 min 90% B and 32–33 min 0% B at a flow rate of 0.2 mL/min and a temperature of 30°C. Isolated GPT (2–5 mg) were dissolved in 1 mL acetonitrile/water (30/70, v/v) acetonitrile containing 0.1% (v/v) TFA and 20 μL were injected. The MS was operated in the positive ionization mode (capillary voltage: -4000 V, end plate offset: -500 V). Nitrogen was used as drying (8.0 L/min, 180°C) and nebulizing gas (0.13 MPa). The scan range was *m/z* 750–3200 (quadrupole ion energy: 5.0 eV). Analysis of the LC-MS data was performed using the software DataAnalysis 3.4 (Bruker Daltonics). M_r_ were calculated with related-ion deconvolution (mass range: 5000–100 000, maximum charge: 100, envelope cut-off: 75%, M_r_ agreement: 0.05%) and maximum entropy deconvolution (mass range: 5000–100 000, instrument resolution power: 10 000).

#### Untargeted LC-MS/MS of chymotryptic GPT hydrolyzates

The isolated GPT (1 mg) were reconstituted in 1 mL of Tris-HCl buffer (0.1 mol/L, pH 7.8, 2 mol urea/L) containing α-chymotrypsin (TLCK treated, ≥ 40 unit/mg protein, Sigma-Aldrich, Steinheim, Germany) at an enzyme/substrate ratio of 1/200 (w/w). After incubation for 24 h at 37°C, the digestion was stopped by addition of 3 μL TFA. The resulting peptide mixtures were subjected to solid phase extraction on Supelco DSC-C_18_ tubes (Sigma-Aldrich). The tubes were conditioned with methanol (1 mL) and equilibrated with TFA (0.1%, v/v, 1 mL). After loading the peptide mixtures, the tubes were washed with water containing TFA (0.1%, v/v, 5 x 1 mL) and the peptides were eluted with methanol (2 mL). The eluate was dried using a vacuum centrifuge (40°C, 6 h, 800 Pa), reconstituted in 500 μL formic acid (FA) (0.1%, v/v), filtered (0.45 μm) and analyzed by ion trap LC-MS/MS [35]. An UltiMate 3000 HPLC system (Dionex) was coupled to an HCTultra PTM ion trap MS (Bruker Daltonics) with collision-induced dissociation (CID). The peptides were separated on an Aeris PEPTIDE XB-C_18_ column (3.6 μm, 10 nm, 2.1 × 150 mm, Phenomenex) and water/FA (999/1, v/v) (A) and acetonitrile/FA (999/1, v/v) (B) as solvents with a flow rate of 0.2 mL/min, a column temperature of 30°C, an injection volume of 10 μL and a linear gradient: 0–5 min 0% B, 45 min 30% B, 55–60 min 90% B, 62–77 min 0% B. The ESI interface was operated using the following parameters: mode: positive, capillary voltage: -4000 V, capillary exit voltage: -1500 V, skimmer voltage: 40 V, drying gas: nitrogen (8.0 L/min, 325°C), nebulizing gas: nitrogen (207 kPa). The MS instrument settings were: scan: standard enhanced, *m/z* range: 500–2000, scan speed: 8.1 *m/z*/s, smart target value: 300 000, maximum acquisition time: 100 ms, MS/MS setting: Auto-MS(n), collision gas: helium, absolute threshold: 10 000, relative threshold: 0.5%, fragmentation amplitude: 0.4 V. Data analysis was carried out with the software DataAnalysis 3.4 and BioTools 3.2 (Bruker Daltonics). A Mascot generic file (*.mgf) was generated from the MS/MS data file, which was exported to the MS/MS ions search module of the Mascot software (Matrix Science, London, UK) using the National Center for Biotechnology Information non-redundant (NCBI) database (U.S. National Library of Medicine, Bethesda, MD, USA) of February 2014. Peptides were searched within the taxonomy *Viridiplantae* with peptide mass tolerance: ± 5 Da, fragment mass tolerance: ± 0.5 Da, mass value: monoisotopic, peptide charges: +1, +2, +3, enzyme: chymotrypsin, maximum number of missed cleavages: 2 and variable modification: ammonia-loss. Peptide ion scores were calculated by the software as -10 × log(P), with P: probability for the observed match being a random event. Peptide scores > 40 were considered to indicate identity or extensive homology (p < 0.05) [36] and scores between 15 and 40 were additionally verified manually [37]. Protein scores (maximum number of protein hits: 30) were derived from peptide scores as sum of the highest ions score for each particular protein sequence, excluding the scores of duplicate matches.

## Results and discussion

The wheat, rye, barley and oat flours were mixtures of four cultivars each to account for the genetic variability between different cultivars [38]. The cultivars were selected based on their production yields relative to the total production of winter wheat, rye, winter and summer barley, and oats in the year 2012 in Germany to include the most relevant cultivars (cumulative production share for wheat: 16%, rye: 53%, barley: 35%, oats: 41%) [39]. For wheat, additional criteria were that the mixture contained flours of three different German baking performance classes (E: elite, A: high, B: bread quality) and covered the most important HMW-GS (cv. Akteur: Ax1, Dx5, Bx7, By9, Dy10; cv. Julius: Ax1, Dx2, Bx6, By8, Dy12; cv. Pamier: Dx5, Bx7, By9, Dy10; cv. Tommi: Dx2, Bx7, By9, Dy12). For rye, three hybrid (cv. Brasetto, cv. Palazzo and cv. Visello) and one population (cv. Conduct) cultivars were chosen. For barley, the selection included two winter (cv. Lomerit, six-row, and cv. Sandra, two-row) and two summer (cv. Grace and cv. Marthe, both two-row) barley cultivars. The contents of water, ash, CP, the Osborne fractions ALGL, prolamins and glutelins as well as gluten were determined for the wheat, rye, barley and oat flours (Table 1) and the quantitative values were in good agreement with earlier studies [26,27,33,40,41].

**Table 1:** Analytical characterization of the flours. Contents of water, ash, crude protein (CP) and the Osborne fractions albumins/globulins (ALGL), prolamins and glutelins of wheat, rye, barley and oat flours (mixture of four cultivars each).

g/100 g of flour	Wheat	Rye	Barley	Oats
Water	13.23 ± 0.17	11.30 ± 0.09	12.85 ± 0.09	11.8 ± 0.16
Ash^a^	0.49 ± 0.01	1.14 ± 0.01	0.87 ± 0.00	1.03 ± 0.00
CP	11.28 ± 0.08	7.13 ± 0.09	7.66 ± 0.10	8.07 ± 0.04
ALGL	1.22 ± 0.01	1.84 ± 0.09	1.24 ± 0.03	2.37 ± 0.04
Prolamins	5.94 ± 0.07	2.53 ± 0.03	3.13 ± 0.06	1.29 ± 0.03
Glutelins	2.98 ± 0.04	0.55 ± 0.01	1.10 ± 0.02	1.01 ± 0.05
Gluten^b^	8.92 ± 0.11	3.08 ± 0.04	4.23 ± 0.08	1.29 ± 0.03^c^
Insoluble residue^d^	1.16 ± 0.05	2.18 ± 0.07	2.23 ± 0.06	3.43 ± 0.04

Values are given as mean ± standard deviation (n = 3) on an as-is basis unless specified

^a^based on dry mass

^b^sum of prolamin and glutelin fractions

^c^only the oat prolamin fraction is considered as oat gluten, because oat glutelins mostly contain 12S globulins [30]

^d^difference between CP and the sum of ALGL, prolamin and glutelin contents quantified by RP-HPLC.

The qualitative RP-HPLC profiles also corresponded well to those reported in the literature [25,27,40], so that all GPT could be assigned within the prolamin and glutelin fractions (Figs 1 and 2). Prolamins were separated into the following GPT: ω5-, ω1,2-, α- and γ-gliadins of wheat (Fig 1A), avenins of oats (Fig 1B), ω-secalins and a minor amount of HMW-secalins, γ-75k- and γ-40k-secalins of rye (Fig 1C and 1D) as well as C- and γ/B-hordeins of barley (Fig 1E and 1F). Glutelins were subdivided into ωb-gliadins, HMW-GS and LMW-GS of wheat (Fig 2A), HMW-, γ-75k- and γ-40k-secalins of rye (Fig 2B) and D- and B/γ-hordeins of barley (Fig 2C).

The separation of rye gluten proteins into prolamins and glutelins according to solubility in ethanol/water (60/40, v/v) was less clear-cut than for wheat [27], so that γ-75k-secalins and a minor part of HMW-secalins appeared in both fractions. Therefore, the content of ω-secalins was calculated from the chromatogram of the unreduced prolamin fraction (Fig 1C). The first peak in the chromatogram of the reduced prolamin fraction contained ω-secalins and a minor amount of HMW-secalins (ωs+H, Fig 1D), so that the difference between ωs+H and ω-secalins alone was due to HMW-secalins. The contents of γ-75k- and γ-40k-secalins were calculated from the respective peak areas in the chromatogram of the reduced prolamin fraction. The percentages given for HMW-, ω-, γ-75k- and γ-40k-secalins (Table 2) are the sum of each rye GPT considering both fractions [27]. The same separation issue was true for barley gluten proteins, because γ- and B-hordeins appeared in both the prolamin and glutelin fractions. Most γ-hordeins are alcohol-soluble monomers, but some form alcohol-insoluble polymers linked by interchain disulfide bonds. The opposite is the case for B-hordeins, some of which are alcohol-soluble monomers, but the majority of which are polymeric [42,43]. It was evident from the chromatogram of the reduced barley prolamins (Fig 1F) that the peak shape of γ/B-hordeins changed after reduction (Fig 1E), while that of the monomeric C-hordeins remained the same. This confirmed that γ/B-hordeins were, at least partly, present as oligomers or polymers linked by disulfide bonds. Unfortunately, the RP-HPLC method applied here did not allow a separation of γ-hordeins from B-hordeins, because there was no separate peak visible in the unreduced prolamin fraction that remained at the same retention time in the reduced prolamin fraction. Due to this limitation, γ- and B-hordeins could only be analyzed and collected together from both fractions. The GPT collected from the prolamin fraction was designated as γ/B-hordeins and that from the glutelin fraction as B/γ-hordeins in the following. Earlier reports found that γ-hordeins are minor components, constituting less than 5% of total hordeins [44–46], which is why this limitation seemed to be acceptable.

**Table 2:** Analytical characterization of the isolated gluten protein types (GPT). Proportions of each GPT in wheat, rye, barley and oat flours, protein content of each isolated GPT, their N-terminal sequence(s), molecular weight ranges (M_r_) determined by LC-ESI-QTOF-MS and the M_r_ of the most appropriate reference sequence found in the NCBI database given with its specific accession.

	Proportion in gluten [%]^a^	Protein content of isolated GPT [%]^a^	N-terminal sequence	M_r_ (LC-ESI-QTOF-MS)	M_r_ (of NCBI accession)^b^	NCBI accession
**Wheat**^**c**^						
HMW-GS^A^	9.3 ± 0.2	94.8 ± 2.5	EGEASGQLQC	83 696^d^	87 643	AHZ62762.1
			EGEASEQLQC		87 256	AHN66476.1
			EGEASRQLQC		68 154	AAU04841.1
ω5-gliadins^B^	5.7 ± 0.2	94.4 ± 3.7	SRLLSPRGKE	48 576 - 54 968	50 927	BAE20328.1
ω1,2-gliadins^C^	7.5 ± 1.0	100.8 ± 1.1	ARELNPSNKE	39 104 - 41 875	39 651	ADA67917.1
α-gliadins^B^	32.6 ± 3.4	88.1 ± 0.7	VRVPVPQLQP	29 994 - 33 979	30 487	AHN85627.1
γ-gliadins^D^	20.8 ± 1.7	92.9 ± 1.1	NMQVDPSGQV	30 295 - 35 212	32 307	P21292.1
LMW-GS^E^	22.3 ± 0.2	81.3 ± 2.1	SHIPGLERPS	32 449 - 41 544	39 478	ACA63857.1
			METSHIPGLE		39 637	ACY08820.1
			METSRVPGLE		37 232	AAP44991.1
**Rye**						
HMW-secalins^A^	5.5 ± 0.3	74.3 ± 3.7	EGEASGQLQC	78 173 - 85 154	78 156	CAC40680.1
γ-75k-secalins^E^	48.5 ± 0.8	94.7 ± 2.7	NMQVNPSGQV	52 313 - 60 476	52 513	ADP95479.1
ω-secalins^C^	18.8 ± 0.6	96.9 ± 4.8	RQLNPSEQEL	39 004 - 39 457	39 359	ACQ83628.1
γ-40k-secalins^D^	27.2 ± 1.3	95.1 ± 4.1	NMQVGPSGQV	32 141 - 32 446	21 377	AEW46799.1
**Barley**						
D-hordeins^A^	7.6 ± 0.2	98.8 ± 3.8	EREINGNNIF	n.d.^d^	72 882	BAA11642.1
C-hordeins^C^	22.7 ± 0.1	95.0 ± 1.0	RQLNPSSQEL	44 786 - 46 722	34 287	AAB28161.1
γ/B-hordeins^D^	51.3 ± 1.4	99.7 ± 1.6	ITTTTMQFNP	31 458 - 34 707	33 168	P80198.1
B/γ-hordeins^E^	18.4 ± 0.5	85.3 ± 6.3	QQQPFPQQPI	31 429 - 34 706	31 444	P06470.1
**Oats**						
avenins		79.2 ± 0.6^e^	TTTVQYNPSE	22 439 - 28 795	23 524	AAA32716.1
			TTTVQYDPSE		23 818	AGB56858.1

^a^Mean ± standard deviation (n = 3) determined by RP-HPLC

^b^monoisotopic mass without signal peptide

^c^proportions of GPT for wheat only add up to 98.2% (not 100%), because ωb-gliadins (1.8%) were not isolated and therefore not included here

^d^only one mass or no masses (n.d.) were detected, because HMW-GS and D-hordeins were difficult to solubilize and ionize

^e^crude protein content (Dumas) of the avenin fraction

^A^homologous high-molecular-weight gluten proteins

^B^unique to wheat

^C^homologous medium-molecular-weight gluten proteins

^D,E^homologous low-molecular-weight gluten proteins. HMW-GS, high-molecular-weight glutenin subunits, LMW-GS, low-molecular-weight glutenin subunits, HMW-secalins, high-molecular-weight secalins.

### Strategy to isolate gluten protein fractions and types

**Fig 3:**
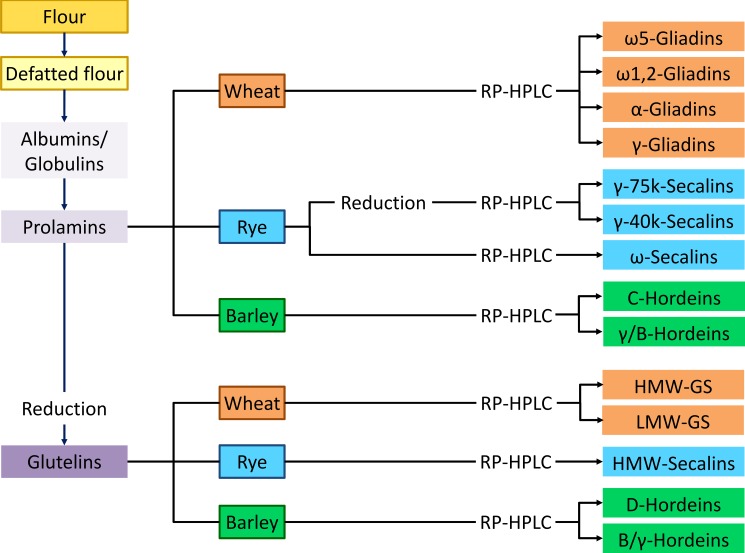
Overview of the preparative strategy. This strategy allows the isolation of well-defined gluten protein fractions and types from wheat, rye, barley and oat flours. HMW-GS, high-molecular-weight glutenin subunits, LMW-GS, low-molecular-weight glutenin subunits.

The analytical characterization of wheat, rye, barley and oat flours was performed with non-defatted flour. For preparative isolation of gluten protein fractions and types it is advisable to use defatted flour [29], especially in the case of oats. A schematic overview of the strategy to prepare defined protein fractions and GPT from wheat, rye and barley flours is presented in Fig 3. Avenins, the prolamin fraction of oats, was not further subdivided. Rye γ-75k- and γ-40k-secalins were only prepared from the reduced prolamin fraction, because the quantities of these GPT were higher in the reduced prolamin fraction than in the glutelin fraction. This procedure is applicable to flours made of pure cultivars as well as to mixtures of cultivars, e.g., as done here with a mixture of four cultivars each, or as described before for the preparation of the PWG-gliadin RM from 28 wheat cultivars [15].

All gluten protein fractions and GPT isolated following this strategy (Fig 3) with yields ranging from 4–7 mg (minor GPT such as ω5- and ω1,2-gliadins and HMW-GS, HMW-secalins and D-hordeins) to 16–36 mg (major GPT such as α-gliadins, LMW-GS, γ-75k-secalins and γ/B-hordeins) per 10 HPLC runs were characterized by determination of the protein content, analytical RP-HPLC, SDS-PAGE, N-terminal sequencing, LC-ESI-QTOF-MS and untargeted LC-MS/MS of chymotryptic GPT hydrolyzates.

### Isolation and characterization of gluten protein fractions and types

#### Wheat (gliadins and glutenins)

**Fig 4:**
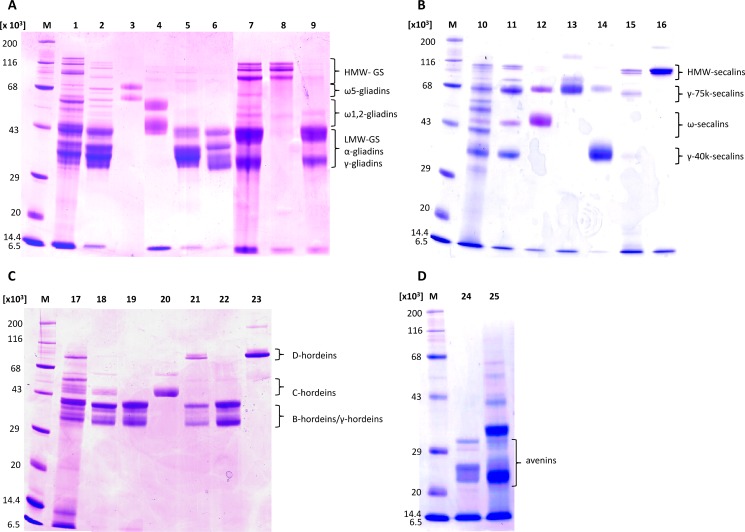
SDS-PAGE of flours, prolamin and glutelin fractions and isolated gluten protein types. (A) Wheat. M: marker, 1: wheat flour, 2: wheat prolamin fraction, 3: ω5-gliadins, 4: ω1,2-gliadins, 5: α-gliadins, 6: γ-gliadins, 7: wheat glutelin fraction, 8: high-molecular-weight glutenin subunits (HMW-GS), 9: low-molecular-weight glutenin subunits (LMW-GS). (B) Rye. M: marker, 10: rye flour, 11: rye prolamin fraction, 12: ω-secalins, 13: γ-75k-secalins, 14: γ-40k-secalins, 15: rye glutelin fraction, 16: HMW-secalins. (C) Barley. M: marker, 17: barley flour, 18: barley prolamin fraction, 19: γ/B-hordeins, 20: C-hordeins, 21: barley glutelins, 22: B/γ-hordeins, 23: D-hordeins. (D) Oats. 24: oat prolamin fraction (avenins), 25: oat flour.

The CP contents of the lyophilized wheat fractions were 93.5 ± 0.4% for gliadins and 82.8 ± 0.2% for glutenins, showing that the extraction procedure from the flour followed by dialysis and lyophilization yielded gluten fractions with very high protein contents comparable to that of PWG-gliadin [15]. The isolated GPT (ω5-, ω1,2-, α- and γ-gliadins and HMW- und LMW-GS) separated from the fractions by preparative RP-HPLC also had very high protein contents ranging from 81.3 ± 2.1% for LMW-GS to 100.8 ± 1.1% for ω1,2-gliadins (Table 2). Re-chromatography of the isolated GPT by analytical RP-HPLC confirmed the identity of each GPT (S1 Fig), because the characteristic retention times matched those in Figs 1A and 2A and there were essentially no impurities visible at 210 nm. SDS-PAGE of the wheat flour, gliadin and glutenin fractions and wheat GPT revealed that all GPT had been obtained in high purity (Fig 4A). The characteristic bands for each GPT were observed at the corresponding M_r_ ranges of 80 000–120 000 for HMW-GS, 60 000–68 000 for ω5-gliadins, 43 000–60 000 for ω1,2-gliadins and 32 000–45 000 for α- and γ-gliadins and LMW-GS, as reported before [31]. Minor traces of HMW-GS (≈ 2.8%, determined by semiquantitative image analysis of the SDS-PAGE gel using ImageJ) were observed in the wheat gliadin fraction, but these disappeared in the HPLC-purified GPT.

N-terminal sequencing of the wheat GPT was used as an additional confirmation of the purity and identity of the isolates (Table 2). Typical N-terminal sequences were determined for all wheat GPT [17,47,48,49], including EGEASGQLQC characteristic of HMW-GS Ax1, Bx7 and Bx6, EGEASEQLQC characteristic of HMW-GS Dx2 and Dx5, and EGEASRQLQC characteristic of HMW-GS By9, Dy10 and Dy12 [31], all of which were present in the wheat flour mixture. For LMW-GS, both the s-type and the m-types [50] were detected by N-terminal sequencing. The M_r_ of the GPT determined by LC-ESI-QTOF-MS were in good agreement with reference sequences in the NCBI database (Table 2). Only one mass signal was detected for HMW-GS, because this GPT was very hard to solubilize. Compared to SDS-PAGE, the M_r_ obtained by LC-ESI-QTOF-MS for the isolated GPT were about 30% lower. This overestimation of M_r_ by SDS-PAGE, which was observed for all GPT studied here, is ascribed to a stretched conformation of the proline-rich sequence domains in the presence of SDS and has frequently been reported before [27,31,51]. Untargeted LC-MS/MS of chymotryptic digests of the single wheat GPT resulted in the identification of 157 characteristic peptides in total. In the hydrolysates of each GPT from wheat, 6 peptides were identified in ω5-gliadins, 24 in ω1,2-gliadins, 31 in α-gliadins, 11 in γ-gliadins, 43 in HMW-GS and 42 in LMW-GS (S1 Table). These peptides matched 12 protein sequences for ω5-gliadins in the NCBI database, 25 for ω1,2-gliadins, 63 for α-gliadins, 28 for γ-gliadins, 64 for HMW-GS and 82 for LMW-GS, all of which had a protein score above 63 (S5 Table), which is the threshold calculated by the Mascot software for a protein identification to be significant. The NCBI accession given in Table 2 as reference sequence for each GPT is the best match considering correct N-terminal sequence, a M_r_ within the range detected by LC-ESI-QTOF-MS and the highest protein score calculated by the Mascot software after untargeted LC-MS/MS analysis considering the type and number of identified peptides. As shown in S5 Table, other protein sequences with higher scores were also assigned to the pool of detected peptides from each GPT, but these either had alternative N-terminal sequences from the main one(s) determined by N-terminal sequencing (e.g., NIQVDPSGQV in AFX69682.1 for γ-gliadins or MENSHIPGLE in ACA63873.1 for LMW-GS) or the sequences in the database were only fragments and not complete protein sequences (e.g., AGK83348.1, AGK83148.1 and AGK83270.1 for LMW-GS). The analysis of the chymotryptic GPT hydrolysates was important to confirm the identities of the isolated GPT, identify characteristic peptides and check for possible impurities. No peptides from other wheat GPT were detected in HMW-GS, ω5-gliadins and ω1,2-gliadins, reconfirming the results of analytical RP-HPLC, SDS-PAGE and N-terminal sequencing. Four and 3 peptides from LMW-GS were detected within the isolated α-gliadins and γ-gliadins, which were assigned to 5 and 9 LMW-GS protein sequences, respectively. Vice-versa, 5 α-gliadin peptides were detected within the LMW-GS isolate that corresponded to 3 α-gliadin accessions (S1 and S5 Tables). Due to their similar M_r_ and RP-HPLC retention times (15 – 20 min, S1C and S1F Fig), α-gliadins and LMW-GS can only be separated according to solubility during sequential extraction of wheat flour. Three extraction steps were shown to yield **≈** 95% of the gliadins [25], but the co-extraction of alcohol-soluble oligomeric HMW gliadins (13–20% of total gliadins) could not be avoided. HMW gliadins consist of **≈** 50% LMW-GS, so that 7–10% of total gliadins are estimated to actually be LMW-GS [32]. To avoid this slight impurity, further pre-fractionation by gel-permeation HPLC would be necessary prior to RP-HPLC, a step which was deemed expendable in the present study after thoroughly weighing benefits (obtaining α-gliadins with > 95% purity as opposed to **≈** 90%) and costs (labor-, material- and time-intensive). Untargeted LC-MS/MS also revealed some additional information, e.g., that LMW-GS of the i-type were also present (e.g., BAB78763.1), which had not been detected by N-terminal sequencing, probably because the i-type occurs in smaller amounts compared to the s- and m-types [50].

#### Rye (secalins)

**Fig 5:**
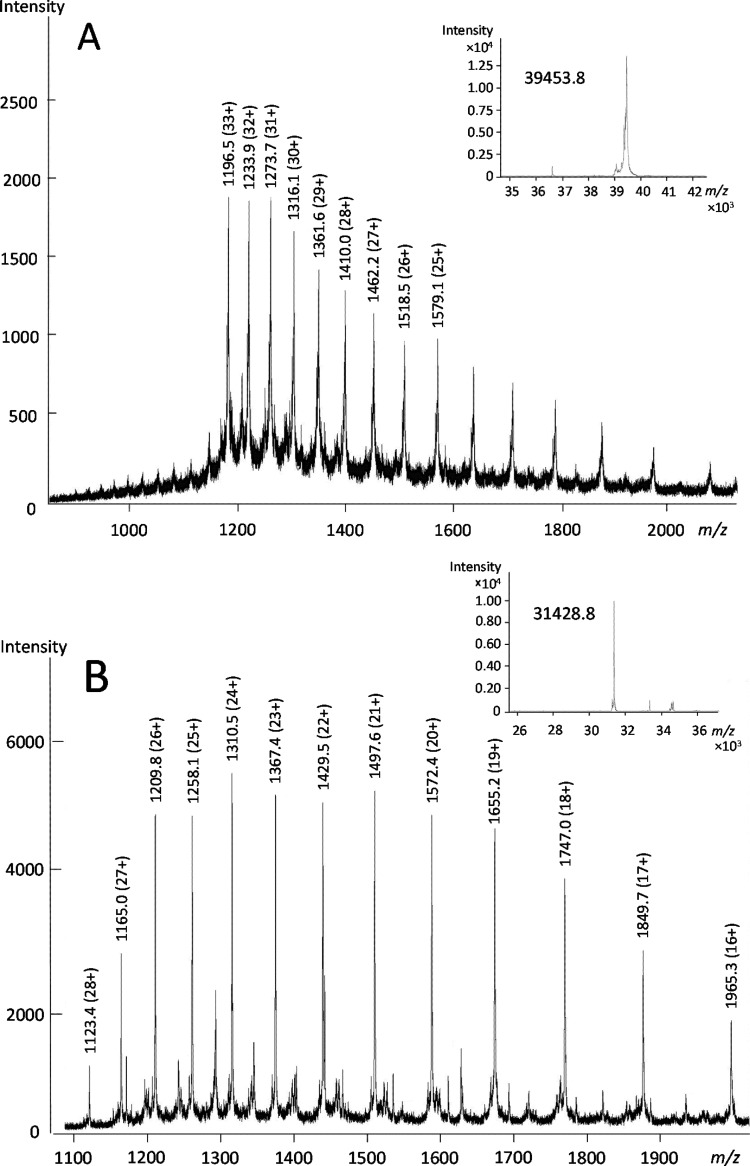
**Mass spectra of isolated (A) ω-secalins and (B) γ-hordeins.** The spectra show the average of scans under the peak with retention times (A) 8.9 min and (B) 12.5 min from the respective base peak MS chromatograms after LC-ESI-QTOF-MS analysis of the isolated ω-secalins and γ-hordeins, respectively. The insets show the mass spectra simulated by maximum entropy deconvolution.

The lyophilized rye prolamin and glutelin fractions had a CP content of 89.4 ± 0.1% and 53.7 ± 0.8%, respectively. The HPLC-purified GPT (HMW-, ω-, γ-75k- and γ-40k-secalins) contained 74.3 ± 3.7% (HMW-secalins) to 96.9 ± 4.8% (ω-secalins) protein (Table 2) and had their identities and purities confirmed by re-chromatography on the analytical RP-HPLC system (S2 Fig) and comparison to Figs 1C and 1D and 2B. The typical M_r_ ranges determined by SDS-PAGE (Fig 4B) were 95 000–105 000 for HMW-secalins, 68 000–75 000 for γ-75k-secalins, 43 000–50 000 for ω-secalins and 35 000–40 000 for γ-40k-secalins, which corresponds well to earlier studies [27]. As already seen with RP-HPLC, the prolamin fraction contained all four secalin types, whereas ω- and γ-40k-secalins were missing from the glutelin fraction. The N-terminal sequence of HMW-secalins was identical to one of the two of wheat x-type HMW-GS, because of the close botanical relationship of wheat and rye [49]. All N-terminal sequences (Table 2) matched those reported in the literature [24,49,51]. LC-ESI-QTOF-MS revealed the M_r_ ranges of all rye GPT (Table 2) and these agreed well with reference sequences for HMW-, γ-75k- and ω-secalins. Fig 5A shows the *m/z*-scans within the peak eluting at 8.9 min that were used for maximum entropy deconvolution to calculate the M_r_ of 39 453.8 of this specific ω-secalin.

In case of γ-40k-secalins, there are only 4 reference sequences in the whole NCBI database (S6 Table), one of which is complete (AEW46799.1), whereas the other 3 are fragments. Even this complete sequence is only predicted and, to the best of our knowledge, there is no reliable reference sequence available, neither in the NCBI nor the UniProtKB database, because the curation status of rye gluten proteins is generally low and there are no reviewed entries (as of December 12, 2016). Some database entries (e.g., ADP95517.1, 75k gamma secalin from *T*. *aestivum*) also appeared to be somewhat imprecisely named, because gluten proteins from *T*. *aestivum* are called gliadins or glutenins, but not secalins. The M_r_ of the γ-40k-secalin entry (21 377, Table 2) was too low compared to the M_r_ determined by LC-ESI-QTOF-MS (≈ 32 300), but this sequence identified by untargeted LC-MS/MS of the chymotryptic γ-40k-secalin digest was the only database match available and identified based on 7 characteristic peptides (γ40k.1, .2, .5, .9, .11, .13 and .15, S2 Table). In total, 78 characteristic rye gluten peptides were identified in the chymotryptic hydrolysates of the single isolated rye GPT, 34 of which were from HMW-secalins, 11 from γ-75k-secalins, 18 from ω-secalins and 16 from γ-40k-secalins (S2 Table). These peptides allowed the identification of 14 protein sequences for HMW-secalins, 65 for γ-75k-secalins, 16 for ω-secalins and 4 for γ-40k-secalins (S6 Table). The extensive homology of wheat and rye gluten proteins [27,49] was again evident from the fact that 3 peptides occurred in both HMW-GS and HMW-secalins (designated HG+HS) and 12 peptides in both ω1,2-gliadins and ω-secalins (designated ωg+ωs). In contrast, the γ-75k- and γ-40k-secalin peptides appeared to be unique to rye. As described above and expected from Gellrich et al. [27], 3 peptides from HMW-secalins (corresponding to 4 protein sequences) were detected in the ω-secalin isolate, but the amount of HMW-secalins is expected to be negligible, because SDS-PAGE of ω-secalins revealed no visible band with an M_r_ of **≈** 100 000 (Fig 4B). No peptides from other rye GPT were detected within HMW-secalins and γ-75k-secalins, but 2 peptides from γ-75k-secalins were detected within the γ-40k-secalin isolate. Due to the virtual lack of reference sequences for γ-40k-secalins, it was impossible to determine whether these 2 peptides (SQLEVVRSL and ASIVTGIVGH) were truly from γ-75k-secalins (which is unlikely, because the RP-HPLC retention times were clearly separated, S2C and S2D Fig), or whether these could also occur in γ-40k-secalins themselves, because both types share the same evolutionary origin [52]. The alignment of both protein sequences (AEW46799.1 and ADP95479.1) using the “Align” tool (UniProtKB) revealed an identity of 30.7%, with homologous sections close to the C-terminus of this γ-75k-secalin sequence. Two very similar peptides (**A**QLEV**I**RSL and AS**T**V**A**GI**G**G**Q**) also occur in this γ-40k-secalin sequence, substantiating the assumption that these peptides could also be present in yet unidentified sequences of γ-40k-secalins, because single to multiple amino acid substitutions occur very frequently within gluten proteins.

#### Barley (hordeins)

The lyophilized barley fractions contained 87.3 ± 0.4% CP (prolamins) and 62.0 ± 0.5% CP (glutelins). The barley GPT (D-, C-, γ- and B-hordeins) isolated by preparative RP-HPLC had protein contents in the range from 85.3 ± 6.3% (B/γ-hordeins) to 99.7 ± 1.6% (γ/B-hordeins) (Table 2). The identities and purities of the GPT were again confirmed by re-chromatography (S3 Fig) and comparison to Figs 1E and 1F and 2C. The separation of the barley prolamin and glutelin fractions and types by SDS-PAGE showed the following M_r_ ≈ 100 000 for D-hordeins, 45 000–65 000 for C-hordeins and 32 000–40 000 for γ- and B-hordeins, which matched earlier investigations [41,53]. The major N-terminal sequences (Table 2) again corresponded to earlier reports [45,47,54] and more specifically matched γ3-hordein and B1-hordein. The M_r_ for γ-hordeins (Fig 5B) and B-hordeins determined by LC-ESI-QTOF-MS were in agreement with the reference sequences from the database, but the M_r_ for C-hordeins (≈ 45 000) was higher than any of the 6 protein sequences that were identified after chymotryptic digestion of the C-hordein isolate and untargeted LC-MS/MS (S3 and S7 Tables). Of those 6 sequences from the NCBI database, 3 were only fragments (P02864.1, P17991.1, AAA32942.1) and the one given as reference sequence (AAB28161.1) already had the highest M_r_ of the remaining 3 entries. Only 9 sequences in total are available for C-hordeins, 5 of which are fragments with a length of 105 amino acids or less (UniProtKB, as of December 12, 2016). The issue of incomplete proteomes within the *Poaceae*, and especially within *Hordeum* sp. and *Secale* sp. has been noted before and often results in unmatched peptide/protein identifications [55]. Overall, the reference sequences identified here for all barley GPT were very similar to those reported by Colgrave et al. [46]. Untargeted LC-MS/MS analyses led to the identification of 45 barley peptides in total, with 9 in the D-hordein, 11 in the C-hordein and 25 in the γ/B- and B/γ-hordein hydrolysates combined, of which 4 were specific for γ-hordeins (S3 Table). One peptide within D-hordeins was also identified in HMW-secalins (KVAKAQQL) and one within B-hordeins also in LMW-GS (LQPHQIAQL). All peptides identified within the C- and D-hordein hydrolysates were specific for that barley GPT, but, as discussed before, the isolation strategy applied here did not allow a separation of γ- and B-hordeins, because both GPT were present in γ/B- and B/γ-hordeins.

#### Oats (avenins)

Oat avenins were extracted from defatted oat flour with 60% ethanol and not further fractionated by preparative HPLC, because this fraction only contained 6 major protein peaks (Fig 1B). Furthermore, only the oat prolamin fraction is considered as oat gluten (avenins), because oat glutelins mostly contain 12S globulins [30] that are not considered as gluten. The CP content of the isolated avenins was 79.2 ± 0.6% and the N-terminal sequence TTTVQYDPSE (Table 2) was found to be similar to ones reported as avenins 5–7 by Anderson [56]. One alternative N-terminal sequence (TTTVQYNPSE) was also detected. The M_r_ range of the avenin fraction was 25 000–32 000 by SDS-PAGE (Fig 4D) and again lower (≈ 22 000–29 000) by LC-ESI-QTOF-MS. The two characteristic bands of α-globulins (≈ 35 000) and β-globulins (≈ 23 000) [30] seen in the oat flour on the SDS-PAGE gel were missing in the avenin fraction. A total of 37 avenin-specific peptides were detected in the chymotryptic hydrolysate by untargeted LC-MS/MS and assigned to 49 avenin protein sequences (S4 and S8 Tables). Globulin-specific peptides were not detected indicating the high purity of the avenin fraction.

## Conclusion

The preparative strategy was suitable to isolate well-defined gluten protein fractions and types from wheat, rye, barley and oat flours in high purity as confirmed by five independent protein analytical methods. The study also highlighted the need for an improvement of the curation status of protein databases within the taxonomy *Poaceae*. Some peptides, especially from C-hordeins, γ-hordeins and γ-40k-secalins, could hardly be matched to corresponding protein sequences or no reference sequence could be found that matched all analytical results, especially considering M_r_ and specific peptide sequences. The isolated GPT may be used as well-defined RM for analytical studies, e.g., for gluten quantitation using targeted LC-MS/MS or for studies on reactivities of antibodies used in ELISA test kits. They may also be applied for clinical studies, e.g., for basophil activation tests in case of wheat allergy, or for a whole variety of other *in vitro* cell- and tissue-based assays to study the mechanisms of CD, NCGS and wheat allergy.

## Supporting information

S1 Fig RP-HPLC chromatograms of the isolated wheat gluten protein types. (A) ω5-gliadins, (B) ω1,2-gliadins, (C) α-gliadins, (D) γ-gliadins, (E) high-molecular-weight glutenin subunits (HMW-GS), (F) low-molecular-weight glutenin subunits (LMW-GS).
(TIF)

S2 Fig RP-HPLC chromatograms of the isolated rye gluten protein types. (A) ω-secalins, (B) high-molecular-weight (HMW)-secalins, (C) γ-75k-secalins, (D) γ-40k-secalins.
(TIF)

S3 Fig RP-HPLC chromatograms of the isolated barley gluten protein types. (A) C-hordeins, (B) γ/B-hordeins, (C) D-hordeins, (D) B/γ-hordeins.
(TIF)

S1 Table Peptides identified in each isolated wheat gluten protein type. Peptide sequences, their scores, *m/z* ratios, charge states and relative molecular weights (M_r_). For corresponding protein sequences, see S5 Table.
(PDF)

S2 Table Peptides identified in each isolated rye gluten protein type. Peptide sequences, their scores, *m/z* ratios, charge states and relative molecular weights (M_r_). For corresponding protein sequences, see S6 Table.
(PDF)

S3 Table Peptides identified in each isolated barley gluten protein type. Peptide sequences, their scores, *m/z* ratios, charge states and relative molecular weights (M_r_). For corresponding protein sequences, see S7 Table.
(PDF)

S4 Table Peptides identified in the oat avenin fraction. Peptide sequences, their scores, *m/z* ratios, charge states and relative molecular weights (M_r_). For corresponding protein sequences, see S8 Table.
(PDF)

S5 Table Protein sequences (protein score > 63) identified in each isolated wheat gluten protein type (GPT). The isolated wheat GPT were digested with chymotrypsin, analyzed by untargeted LC-MS/MS and the MS/MS files searched using the Mascot software and the NCBI Protein database (taxonomy *Viridiplantae*).
(PDF)

S6 Table Protein sequences (protein score > 63) identified in each isolated rye gluten protein type (GPT). The isolated rye GPT were digested with chymotrypsin, analyzed by untargeted LC-MS/MS and the MS/MS files searched using the Mascot software and the NCBI Protein database (taxonomy *Viridiplantae*).
(PDF)

S7 Table Protein sequences (protein score > 63) identified in each isolated barley gluten protein type (GPT). The isolated barley GPT were digested with chymotrypsin, analyzed by untargeted LC-MS/MS and the MS/MS files searched using the Mascot software and the NCBI Protein database (taxonomy *Viridiplantae*).
(PDF)

S8 Table Protein sequences (protein score > 63) identified in oat avenins. The isolated avenin fraction was digested with chymotrypsin, analyzed by untargeted LC-MS/MS and the MS/MS files were searched using the Mascot software and the NCBI Protein database (taxonomy *Viridiplantae*).
(PDF)

## References

[1] FAOSTAT. Food and Agriculture Organization of the United Nations, Statistics Division. Available: http://faostat3.fao.org/browse/Q/QC/E (accessed December 12, 2016).

[2] Sapone A, Bai JC, Ciacci C, Dolinsek J, Green PH, Hadjivassiliou M, et al Spectrum of gluten-related disorders: consensus on new nomenclature and classification. BMC Med. 2012; 10: 13 10.1186/1741-7015-10-13 22313950 PMC3292448

[3] Ludvigsson JF, Leffler DA, Bai JC, Biagi F, Fasano A, Green PH, et al The Oslo definitions for coeliac disease and related terms. Gut. 2013; 62: 43–52. 10.1136/gutjnl-2011-301346 22345659 PMC3440559

[4] Scherf KA, Koehler P, Wieser H. Gluten and wheat sensitivities–an overview. J Cereal Sci. 2016; 67: 2–11.

[5] Tatham AS, Shewry PR. Allergens in wheat and related cereals. Clin Exp Allergy. 2008; 38: 1712–1726. 10.1111/j.1365-2222.2008.03101.x 18823308

[6] Fasano A, Sapone A, Zevallos V, Schuppan D. Nonceliac gluten sensitivity. Gastroenterology. 2015; 148: 1195–1204. 10.1053/j.gastro.2014.12.049 25583468

[7] Wieser H, Koehler P, Konitzer K. Celiac disease and gluten—multidisciplinary challenges and opportunities 1st ed. Amsterdam, New York, San Diego: Academic Press Elsevier; 2014.

[8] Dupont FM, Vensel WH, Tanaka CK, Hurkman WJ, Altenbach SB. Deciphering the complexities of the wheat flour proteome using quantitative two-dimensional electrophoresis, three proteases and tandem mass spectrometry. Proteome Sci. 2011; 9: 10 10.1186/1477-5956-9-10 21314956 PMC3238214

[9] Camarca A, Del Mastro A, Gianfrani C. Repertoire of gluten peptides active in celiac disease patients: perspectives for translational therapeutic applications. Endocr Metab Immune Disord Drug Targets. 2012; 12: 207–219. 22385112 10.2174/187153012800493549

[10] Stoven S, Murray JA, Marietta EV. Latest in vitro and in vivo models of celiac disease. Expert Opin Drug Discov. 2013; 8: 445–457. 10.1517/17460441.2013.761203 23293929 PMC3605231

[11] Takahashi H, Matsuo H, Chinuki Y, Kohno K, Tanaka A, Maruyama N, Morita E. Recombinant high molecular weight-glutenin subunits-specific IgE detection is useful in identifying wheat-dependent exercise-induced anaphylaxis complementary to recombinant omega-5 gliadin-specific IgE test. Clin Exp Allergy. 2012; 42: 1293–1298. 10.1111/j.1365-2222.2012.04039.x 22805477

[12] Brockow K, Kneissl D, Valentini L, Zelger O, Grosber M, Kugler C, et al Using a gluten oral food challenge protocol to improve diagnosis of wheat-dependent exercise-induced anaphylaxis. J Allergy Clin Immunol. 2015; 135: 977–987.e4 10.1016/j.jaci.2014.08.024 25269870

[13] Dewar DH, Amato M, Ellis HJ, Pollock EL, Gonzalez-Cinca N, Wieser H, Ciclitira PJ. The toxicity of high molecular weight glutenin subunits of wheat to patients with coeliac disease. Eur J Gastroenterol Hepatol. 2006; 18: 483–491. 16607142 10.1097/00042737-200605000-00005

[14] Scherf KA, Poms RE. Recent developments in analytical methods for tracing gluten. J Cereal Sci. 2016; 67: 112–122.

[15] Van Eckert R, Berghofer E, Ciclitira PJ, Chirdo F, Denery-Papini S, Ellis HJ, et al Towards a new gliadin reference material—isolation and characterisation. J Cereal Sci. 2006; 43: 331–341.

[16] Schwalb T, Wieser H, Koehler P. Comparison of different protein references and ELISA kits for the detection of gluten in foods. In: Stern M, editor. Proceedings of the 24th workshop on prolamin analysis and toxicity. Zwickau (Germany): Verlag Wissenschaftliche Scripten; 2011 pp. 23–29.

[17] Arentz-Hansen H, McAdam SN, Molberg Ø, Kristiansen C, Sollid LM. Production of a panel of recombinant gliadins for the characterisation of T cell reactivity in coeliac disease. Gut. 2000; 46: 46–51. 10.1136/gut.46.1.46 10601054 PMC1727782

[18] Arentz-Hansen H, Körner R, Molberg Ø, Quarsten H, Vader W, Kooy YMC, et al The intestinal T cell response to α-gliadin in adult celiac disease is focused on a single deamidated glutamine targeted by tissue transglutaminase. J Exp Med. 2000; 191: 603–612. 10684852 10.1084/jem.191.4.603 PMC2195837

[19] Srinivasan B, Focke-Tejkl M, Weber M, Pahr S, Baar A, Atreya R, et al Usefulness of recombinant γ-gliadin 1 for identifying patients with celiac disease and monitoring adherence to a gluten-free diet. J Allergy Clin Immunol. 2015; 136: 1607–1618.e3 10.1016/j.jaci.2015.04.040 26078104 PMC4669310

[20] Ellis HJ, Lozano-Sanchez P, Bermudo-Redondo C, Suligoj T, Biagi F, Bianchi PI, et al Antibodies to wheat high-molecular-weight glutenin subunits in patients with celiac disease. Int Arch Allergy Immunol. 2011; 159: 428–434. 10.1159/000338284 22813868

[21] Matsuo H, Kohno K, Morita E. Molecular cloning, recombinant expression and IgE-binding epitope of ω5-gliadin, a major allergen in wheat-dependent exercise-induced anaphylaxis. FEBS Journal. 2005; 272: 4431–4438. 10.1111/j.1742-4658.2005.04858.x 16128812

[22] ICC Standard No. 110/1. Determination of the moisture content of cereals and cereal products (practical method). International Association for Cereal Science and Technology. 1976.

[23] ICC Standard No. 104/1. Determination of ash in cereals and cereal products. International Association for Cereal Science and Technology. 1990.

[24] ICC Standard No. 167. Determination of crude protein in grain and grain products for food and feed by the Dumas combustion principle. International Association for Cereal Science and Technology. 2000.

[25] Wieser H, Antes S, Seilmeier W. Quantitative determination of gluten protein types in wheat flour by reversed-phase high-performance liquid chromatography. Cereal Chem. 1998; 75: 644–650.

[26] Lexhaller B, Tompos C, Scherf KA. Comparative analysis of prolamin and glutelin fractions from wheat, rye and barley with five sandwich ELISA test kits. Anal Bioanal Chem. 2016; 408: 6093–6104. 10.1007/s00216-016-9721-7 27342795

[27] Gellrich C, Schieberle P, Wieser H. Biochemical characterization and quantification of the storage protein (secalin) types in rye flour. Cereal Chem. 2003; 80: 102–109.

[28] Scherf KA. Impact of the preparation procedure on gliadin, glutenin and gluten contents of wheat starches determined by RP-HPLC and ELISA. Eur Food Res Technol. 2016; 242: 1837–1848.

[29] Pflaum T, Konitzer K, Hofmann T, Koehler P. Analytical and sensory studies on the release of sodium from wheat bread crumb. J Agric Food Chem. 2013; 61: 6485–6494. 10.1021/jf4012906 23799642

[30] Robert LS, Nozzolillo C, Altosaar I. Characterization of oat (*Avena sativa* L.) residual proteins. Cereal Chem. 1985; 62: 276–279.

[31] Lagrain B, Rombouts I, Wieser H, Delcour JA, Koehler P. A reassessment of the electrophoretic mobility of high molecular weight glutenin subunits of wheat. J Cereal Sci. 2012; 56: 726–732.

[32] Schmid M, Wieser H, Koehler P. Isolation and characterization of high-molecular-weight (HMW) gliadins from wheat flour. Cereal Chem. 2016; 93: 536–542.

[33] Koenig A, Konitzer K, Wieser H, Koehler P. Classification of spelt cultivars based on differences in storage protein compositions from wheat. Food Chem. 2015; 168: 176–182. 10.1016/j.foodchem.2014.07.040 25172697

[34] Lagrain B, Brunnbauer M, Rombouts I, Koehler P. Identification of intact high molecular weight glutenin subunits from the wheat proteome using combined liquid chromatography-electrospray ionization mass spectrometry. PLOS ONE. 2013; 8: e58682 10.1371/journal.pone.0058682 23520527 PMC3592795

[35] Scherf KA, Wieser H, Koehler P. Improved quantitation of gluten in wheat starch for celiac disease patients by gel-permeation high-performance liquid chromatography with fluorescence detection (GP-HPLC-FLD). J Agric Food Chem. 2016; 64: 7622–7631. 10.1021/acs.jafc.6b02512 27633005

[36] Rombouts I, Lagrain B, Brunnbauer M, Delcour JA, Koehler P. Improved identification of wheat gluten proteins through alkylation of cysteine residues and peptide-based mass spectrometry. Sci Rep. 2013; 3: 2279 10.1038/srep02279 23880742 PMC3721084

[37] Chen Y, Kwon SW, Kim SC, Zhao Y. Integrated approach for manual evaluation of peptides identified by searching protein sequence databases with tandem mass spectra. J Proteome Res. 2005; 4: 998–1005. 10.1021/pr049754t 15952748

[38] Hajas L, Scherf KA, Bugyi Z, Török K, Schall E, Köhler P, Tömösközi S. ELISA response and gliadin composition of different wheat varieties grown in multiple harvest years. Acta Aliment. 2016.

[39] Bundesministerium für Ernährung, Landwirtschaft und Verbraucherschutz (BMELV), Besondere Ernte- und Qualitätsermittlung 2012. http://berichte.bmelv-statistik.de/EQB-1002000-2012.pdf

[40] Eggert K, Wieser H, Pawelzik E. The influence of Fusarium infection and growing location on the quantitative protein composition of (Part II) naked barley (*Hordeum vulgare nudum*). Eur Food Res Technol. 2010; 230: 893–902.

[41] Lange M, Vincze E, Wieser H, Schjoerring JK, Holm PB. Suppression of C-hordein synthesis in barley by antisense constructs results in a more balanced amino acid composition. J Agric Food Chem. 2007; 55: 6074–6081. 10.1021/jf0709505 17580876

[42] Shewry PR, Tatham AS. The prolamin storage proteins of cereal seeds: structure and evolution. Biochem J. 1990; 267: 1–12. 2183790 10.1042/bj2670001 PMC1131235

[43] Celus I, Brijs K, Delcour JA. The effects of malting and mashing on barley protein extractability. J Cereal Sci. 2006; 44: 203–211.

[44] Shewry PR, Kreis M, Parmar S, Lew, EJ-L, Kasarda DD. Identification of γ-type hordeins in barley. FEBS Lett. 1985; 190: 61–64.

[45] Rechinger KB, Simpson DJ, Svendsen I, Cameron-Mills V. A role for γ3 hordein in the transport and targeting of prolamin polypeptides to the vacuole of developing barley endosperm. Plant J. 1993; 4: 841–853. 7506098 10.1046/j.1365-313x.1993.04050841.x

[46] Colgrave ML, Goswami H, Howitt CA, Tanner GJ. What is in a beer? Proteomic characterization and relative quantification of hordein (gluten) in beer. J Proteome Res 2012; 11: 386–396. 10.1021/pr2008434 21999962

[47] Tatham AS, Shewry PR. The S-poor prolamins of wheat, barley and rye: revisited. J Cereal Sci. 2012; 55: 79–99.

[48] Lew EJ-L, Kuzmicky DD, Kasarda DD. Characterization of low molecular weight glutenin subunits by reversed-phase high-performance liquid chromatography, sodium dodecyl sulfate-polyacrylamide gel electrophoresis, and N-terminal amino acid sequencing. Cereal Chem. 1992; 69: 508–515.

[49] Kasarda DD, Autran J-C, Lew EJ-L, Nimmo CC, Shewry PR. N-terminal amino acid sequences of ω-gliadins and ω-secalins. Implications for the evolution of prolamin genes. Biochim Biophys Acta. 1983; 747: 138–150.

[50] Lee J-Y, Beom H-R, Altenbach SB, Lim S-H, Kim Y-T, Kang C-S, et al Comprehensive identification of LMW-GS genes and their protein products in a common wheat variety. Funct Integr Genomics. 2016; 16: 269–279. 10.1007/s10142-016-0482-3 26882917

[51] Rocher A, Calero M, Soriano F, Méndez M. Identification of major rye secalins as coeliac immunoreactive proteins. Biochim Biophys Acta. 1996; 1295: 13–22. 8679669 10.1016/0167-4838(95)00269-3

[52] Shewry PR, Field JM. The purification and characterization of two groups of storage proteins (secalins) from rye (*Secale cereale* L.). J Exp Bot. 1982; 33: 261–268.

[53] Tanner GJ, Blundell MJ, Colgrave ML, Howitt CA. Quantification of hordeins by ELISA: the correct standard makes a magnitude of difference. PLOS ONE. 2013; 8: e56456 10.1371/journal.pone.0056456 23509607 PMC3585327

[54] Pistón F, Shewry PR, Barro F. D hordeins of *Hordeum chilense*: a novel source of variation for improvement of wheat. Theor Appl Genet. 2007; 115: 77–86. 10.1007/s00122-007-0542-0 17458535

[55] Colgrave ML, Goswami H, Howitt CA, Tanner GJ. Proteomics as a tool to understand the complexity of beer. Food Res Int 2013; 54: 1001–1012.

[56] Anderson OD. The spectrum of major seed storage genes and proteins in oats (Avena sativa). PLOS ONE 2014; 9: e83569 10.1371/journal.pone.0083569 25054628 PMC4108316

