# Supplementary material for: Isolation and characterization of gluten protein types from wheat, rye, barley and oats for use as reference materials
Source: PLoS One. 2017 Feb 24;12(2):e0172819. doi: 10.1371/journal.pone.0172819 (PMC5325591; doi:10.1371/journal.pone.0172819)
Supplement: S1 Table — Peptide sequences, their scores, m/z ratios, charge states and relative molecular weights (Mr). For corresponding protein sequences, see S5 Table. (PDF) [file pone.0172819.s004.pdf]

S1 Table. Peptides identified in each isolated wheat gluten protein type. Peptide sequences, their scores, *m/z* ratios, charge states and relative molecular weights (M_r). For corresponding protein sequences, see S5 Table.

Abbre-viation	Gluten protein type Peptide sequence	Peptide score	*m/z* (charge state)	M_r
	**HMW-GS**			
HG.1	YPTSLQQTGQGQQSGQGQQGYY	62	802.49 (+3)	2403.08
HG.2	LQPGQGQQGY	58	538.16 (+2)	1074.52
HG.3	YPGQASSQRPGQGQQPGQGQQEY	56	826.32 (+3)	2475.12
HG.4	QQTGQGQQSGQGQQGYY	56	921.92 (+2)	1841.80
HG.5	QPEQLQQPTQGQQRQQPGQGQQL	55	877.73 (+3)	2629.30
HG.6	WQPGQGQQGY	54	574.72 (+2)	1147.51
HG.7	YLTSPQQSGQW	53	647.78 (+2)	1293.61
HG.8	YLSVTSPQQVSY	51	686.69 (+2)	1370.68
HG.9	SSSLQQPGQGLQGHYPASL	51	652.60 (+3)	1953.96
HG.10	TASLQQPGQGQQGHYPASL	46	656.94 (+3)	1966.96
HG.11	YPGQASSQRPGQGQQPGQGQQEYY	46	880.43 (+3)	2638.19
HG.12	QLGQGQQGY	43	489.71 (+2)	977.46
HG+HS.1	QQPGQGQQPGQW	42	669.79 (+2)	1337.62
HG.13	LQPGQGQQGYYPTSL	41	819.26 (+2)	1635.80
HG.14	YYPTSPQESGQGQQPGQW	41	1019.84 (+2)	2036.89
HG.15	STSLQQPGQGQQGHYPASL	41	992.96 (+2)	1982.95
HG.16	QQTGQGQQSGQGQQGY	41	840.61 (+2)	1678.74
HG.17	SQPRQGQQGY	40	574.72 (+2)	1147.54
HG+HS.2	HVSAEHQAASL	40	575.34 (+2)	1148.56
HG.18	LSVTSPQQVSY	39	604.73 (+2)	1207.62
HG.19	SSYHVSVEHQAASL	39	757.95 (+2)	1513.72
HG.20	QQPGQGQPGYY	38	611.76 (+2)	1221.55
HG.21	TTSLQQSGQGQQGY	36	741.88 (+2)	1481.68
HG.22	QQPGQGQQGHYPASL	35	798.39 (+2)	1594.76
HG.23	QQPGQGQQGHY	35	798.39 (+2)	1226.55
HG.24	HQSGQGQQGYYPSTL	34	825.85 (+2)	1649.75
HG.25	KRYYPSVTSPQQVSY	34	902.29 (+2)	1801.91
HG+HS.3	HVSVEHQAASL	33	589.34 (+2)	1176.60
HG.26	TASLQQPGQGQQGHYPASLQQVGQGQQIGQL	33	1078.65 (+3)	3231.61
HG.27	QQPGQWQQPGQGQPGYY	32	974.29 (+2)	1945.88
HG.28	QQSGQGQPGYYPTSL	30	805.97 (+2)	1609.74
HG.29	GTSSQTVQGYYPSVSSPQQGPY	30	1160.03 (+2)	2317.06
HG.30	HQSGQGQQGYYPSSL	29	818.88 (+2)	1635.74
HG.31	EQQPVVPSKAGSFYPSETTPSQQL	26	1303.66 (+2)	2604.28
HG.32	YPSITSPQQVSY	25	686.69 (+2)	1368.66
HG.33	KACQQVMDQQLRDISPECHPVVVSPVAGQY	25	1108.59 (+3)	3324.61
HG.34	QQSGQGQHGYYPTSPQLSGQGQRPGQW	23	986.61 (+3)	2956.37
HG.35	YPGQASPQQPGQGQQPGKW	22	1020.20 (+2)	2037.97
HG.36	EQTVVPPKGGSFYPGETTPL	20	702.39 (+3)	2103.06
HG.37	EQQIVVPPKGGSFYPGETTPPQQL	20	866.87 (+3)	2596.32
HG.38	QQSGQGQQGYYPTSPQQSGQGQQPGQW	19	979.64 (+3)	2934.30
HG.39	QQSGQGQQGHYPTSLQQPGQGQQGHY	18	942.45 (+3)	2823.28
HG.40	LQPGQGQQGYYPTSPQQPGQGQQL	16	863.56 (+3)	2584.24

	**ω5-gliadins**			
ω5.1	QQYPQQQPSGSDVISISGL	53	677.95 (+3)	2031.00
ω5.2	QQYPQQQPSGSNVISISGL	33	677.95 (+3)	2030.01
ω5.3	QTPQEQFPQEQFPEPQQILQQQF	33	948.38 (+3)	2842.36
ω5.4	SPRGMELQTPQEQFPQEQFPEPQQILQQQF	32	1205.20 (+3)	3612.74
ω5.5	SPRGMELQTPQEQFPQEQFPEPQQIL	27	1028.40 (+3)	3081.49
ω5.6	HQQQLPQQQFPQQQFPQQQFPQQQQFPQQQQL	15	1340.10 (+3)	4015.95
	**ω1,2-gliadins**			
ωg.1	ARELNPSNKELQSPQQSF	53	1036.76 (+2)	2072.04
ωg+ωs.1	GQQPEQIISQRPQQPFPLQPQQQF	50	950.27 (+3)	2846.45
ωg.2	GSSLTSIGGQ	48	453.58 (+2)	905.45
ωg+ωs.2	GEQPEQIISQRPQQPFPLQPQQPF	44	939.61 (+3)	2816.43
ωg+ωs.3	AQQPEQIISQQPFPLQPQQPF	43	1224.98 (+2)	2448.25
ωg+ωs.4	AQQPEQLISQQPFPLQPQQPF	43	1224.98 (+2)	2448.25
ωg.3	QQPQQQL	42	435.11 (+2)	868.45
ωg+ωs.5	GQQPEQIISQRPQQPFPLQPKQPF	39	939.77 (+3)	2815.48
ωg+ωs.6	GQQPEQIISQRPQQPFPLQPQQPF	37	1408.84 (+2)	2815.45
ωg.4	LRQSQQPF	36	502.16 (+2)	1002.53
ωg+ωs.7	GQQPMQIISQRPQQPFPLQPQQPF	34	938.83 (+3)	2817.45
ωg.5	AQQPEQIISHQPFPLQPQQPF	33	820.08 (+3)	2457.25
ωg+ωs.8	SLQPQQPVPQQPQRPF	32	937.78 (+2)	1873.99
ωg+ωs.9	SQPQQPFPQQPGQIIPEQPQQPSPLQPQQPF	30	1173.74 (+3)	3518.77
ωg.6	YQPQQPFPQQPGQIIPQQPQQPSPLQPQQPF	29	1199.47 (+3)	3593.81
ωg.7	FPHQSQQPF	26	558.10 (+2)	1114.53
ωg.8	SQQPQRPQQPFPQQPQQIIPPQPQQPF	23	1064.69 (+2)	3191.63
ωg.9	SQPQQPF	23	831.70 (+1)	830.40
ωg.10	LQQPQQPSPQPQQVVQIIPQQPQQPFPL	22	1072.63 (+3)	3215.72
ωg.11	GRQPEQIISQRPQQPFPLQPQQPF	22	950.27 (+3)	2843.49
ωg+ωs.10	SQPQQPFPQQPGQIIPQQPQQPSPLQPQQPF	18	1173.74 (+3)	3517.78
ωg+ωs.11	SQPQQPFPQQPGQIIPKQPQQPSPLQPQQPF	17	1173.95 (+3)	3517.82
ωg+ωs.12	GQQPEQIISRRPQQPFPLQPQQPF	17	949.97 (+3)	2843.49
ωg.12	SQQPEQTISQQPQQPFPQQPHQPQQPYPQQQPY	15	1314.06 (+3)	3939.86
	**α-gliadins**			
α.1	GSSQVLQQSTY	63	599.15 (+2)	1196.57
α.2	FQPSQQNPQAQGF	57	738.70 (+2)	1475.69
α.3	RQQQKQQQQPSSQVSF	56	644.59 (+3)	1930.97
α.4	LQLQPFPQPQLPYPQPQPF	52	784.80 (+2)	2262.19
α.5	LQLQPFPQPQLPY	52	784.80 (+2)	1567.85
α.6	RPQQPYPQPQPQY	45	813.71 (+2)	1625.80
α.7	LQLQPFPQPQLPYPQPQLPYPQPQPF	45	1029.75 (+3)	3085.61
α.8	QQPQQQYPLGQGSF	39	803.16 (+2)	1604.77
α.9	MQLQPFPQPQLPYPQPQLPYPQPQPF	37	1035.76 (+3)	3103.57
α.10	QQPQQQYPSGQGF	36	746.68 (+2)	1491.68
α.11	LGQQQPFPPQQPYPQPQPFPSQQPY	35	974.63 (+3)	2921.42
α.12	IGQQQPFPPQQPYPQPQPFPSQQPY	35	974.63 (+3)	2921.42
α.13	LGQEQPFPPQQPYPQPQPFPSQQPY	33	974.63 (+3)	2922.41
α.14	VQQQQFPGRQQPFAPQQPYPQPQPFPSQQPY	31	1222.66 (+3)	3665.79
α.15	RGQQQPFPPQQPYPQPQPFPSQQPY	30	988.86 (+3)	2964.44
α.16	LGQQQPFTPQQPYPQPQPFPSQQPY	28	974.63 (+3)	2925.42
α.17	HQQRQQPSSQVSF	27	519.53 (+3)	1555.75

α.18	LQLQPFPQPQLPYPQPQLPYPQPQLPYPQPQPF	27	1304.40 (+3)	3909.04
α.19	LGQQQTFPPQQPYPQPQPFPSQQPY	26	974.63 (+3)	2925.42
α.20	VRVPVPQLQPQNPSQQQPQEQVPL	26	912.53 (+3)	2733.46
α.21	VQQQQFVGQQQPFPPQQPYPQPQPFPSQQPY	25	1222.27 (+3)	3665.78
α.22	QQPQQQYPSGQGFFQPSQQNPQAQGF	23	984.15 (+3)	2949.35
α.23	LGQQQQQFPGQQQPFPPQQPYPQPQPF	23	1054.99 (+3)	3162.54
α.24	VQEQQFPGQQQPFPPQQPYPQPQPFPSQQPY	23	1222.33 (+3)	3664.74
α.25	QQPQQQYPSGQGSFQPSQQNPQAQGF	23	964.16 (+3)	2889.31
α.26	NGQQQPFPPQQPYPQPQPFPSQQPY	22	974.96 (+3)	2922.38
α.27	LGQQQPFPPQQPYPQPQFPSQLPYL	21	974.63 (+3)	2922.48
α.28	VQQQQFPGQQQPFPPQQPYPQPQPFPSQQPY	20	1222.27 (+3)	3663.76
α.29	LQLQPFPQPQLPYPHPQLPYPQPQPF	21	1032.72 (+3)	3094.61
α.30	VRVTVPQLQPQNPSQQQPQEQVPL	16	912.53 (+3)	2737.46
α.31	LQLQPFPQTQLPY	15	784.89 (+2)	1571.84
	**LMW-GS within α-gliadins**			
LG.1	SHHQQQQPIQQQPQPF	56	652.53 (+3)	1954.95
LG.2	SQQQQIVL	31	943.20 (+1)	942.52
LG.3	VLPQQQIPF	29	535.17 (+2)	1068.60
LG.4	GVGTGVGSY	20	796.13 (+1)	795.38
	**γ-gliadins**			
γg.1	LQPQQPQQSFPQQQQPL	67	1010.32 (+2)	2019.02
γg.2	NIQVDPSGQVQW	54	685.74 (+2)	1369.67
γg.3	QLVQGQGIIQPQQPAQY	48	632.75 (+3)	1895.00
γg.4	ANIDAGIGGQ	45	915.29 (+1)	914.45
γg.5	FQLVQGQGIIQPQQPAQY	43	681.65 (+3)	2042.07
γg.6	ASIVAGIGGQ	42	436.72 (+2)	871.48
γg.7	SQQPQQAF	42	467.13 (+2)	932.44
γg.8	NIQVDPSGQVQWPQQQPFPQPQPF	42	930.65 (+3)	2789.36
γg.9	SQQQQVGQGSLVQGQGIIQPQQPAQL	38	915.95 (+3)	2744.43
γg.10	QVQWPQQQPFPQPQQPF	29	1054.77 (+2)	2107.03
γg.11	RQPQQPF	25	450.64 (+2)	899.47
	**LMW-GS within γ-gliadins**			
LG.1	SHHQQQQPIQQQPQPF	56	652.45 (+3)	1954.95
LG.2	SQQQQIVL	46	943.45 (+1)	942.52
LG.3	VLPQQQIPF	35	535.25 (+2)	1068.60
	**LMW-GS**			
LG.5	SIILQEQQQGF	68	645.90 (+2)	1289.67
LG.6	QQQLGKQPQQQQL	65	776.36 (+2)	1550.82
LG.7	QQQLGQQPQQQKL	65	776.36 (+2)	1550.82
LG.8	QQQLGQQPQQQQL	65	776.36 (+2)	1550.79
LG.9	GQQPQQQQLAQGTF	63	779.94 (+2)	1557.76
LG.10	SIVLQEQQHGQGL	61	718.92 (+2)	1435.75
LG.11	SQQQPPFSQQQQQPL	56	885.31 (+2)	1767.86
LG.1	SHHQQQQPIQQQPQPF	56	652.80 (+3)	1954.95
LG.12	QQPQQQLGQQPQQQQQQVL	55	754.33 (+3)	2259.14
LG.13	SIVLQEQQHGQGF	53	736.22 (+2)	1469.73
LG.14	QQPQQQLGQQPQQQVQKGTF	51	1227.1 (+2)	2451.23
LG.15	VQPQQQQPQQSVQGVY	50	921.88 (+2)	1842.91
LG.16	GKQPQQQQL	45	527.74 (+2)	1053.56
LG.17	GQQPQQQKL	45	527.74 (+2)	1053.56
LG.18	GQQPQQQQL	45	527.74 (+2)	1053.56
LG.19	LQPHQIAQLEVMNSIAL	44	953.19 (+2)	1904.03

LG.20	SILVQEQQHGQGL	44	718.92 (+2)	1435.75
LG.21	QQPQQQLGQQPQEQQVQQGTF	44	818.47 (+3)	2452.18
LG.22	QQQPLPPQQTFPQQPL	41	938.44 (+2)	1873.98
LG.23	SIILQQQQQQQQQQQGQSIIQY	41	882.21 (+3)	2642.35
LG.3	VLPQQQIPF	37	535.31 (+2)	1068.60
LG.24	SHIPGLERPSQQQPLPPQQTL	37	784.65 (+3)	2350.25
LG.25	SQQQQIPVIHPSVL	35	525.31 (+3)	1572.87
LG.26	LQPHQIAQLEVMTSIAL	35	946.59 (+2)	1891.03
LG.27	LQPHKIAQLEVMTSIAL	35	946.59 (+2)	1891.07
LG.28	SQQQQPPFSQQQPPF	33	886.86 (+3)	1770.84
LG.29	SQIPEQSRY	33	554.28 (+2)	1106.54
LG+B	LQPHQIAQL	32	524.31 (+2)	1046.59
LG.31	LQPHKIAQL	32	524.31 (+2)	1046.63
LG.32	DAIRAIIY	31	467.77 (+2)	933.53
LG.33	EAIRAIIFSIILQEQQQGF	27	735.84 (+3)	2203.21
LG.34	SQHQQPVLPQQQIPY	26	895.07 (+2)	1789.92
LG.35	IQVDPSGQVQWPQQQQPFPQPQQPF	25	978.39 (+3)	2931.44
LG.36	SQQQPPFSQQQQPPF	23	885.31 (+2)	1770.84
LG.37	VQQQLPVVQPSIL	23	725.27 (+2)	1447.85
LG.38	VQQQIPVVQPSIL	23	725.27 (+2)	1447.85
LG.39	SIILQQQQQQQQQQQQQGQSIIQY	23	1010.32 (+3)	3026.52
LG.40	TQQQPPFSQQPPISQQQQQQQQQQQPF	22	1079.74 (+3)	3234.55
LG.41	SQQQQPPFSQQQPPFSQQQQQPL	19	1355.76 (+2)	2708.30
	**α-gliadins within LMW-GS**			
α.32	QQQQQQQQQQIL	78	827.72 (+2)	1652.83
α.2	FQPSQQNPQAQGF	48	738.95 (+2)	1475.69
α.33	VQPQQLPQFEEIRNL	41	920.46 (+2)	1837.98
α.10	QQPQQQYPSGQGF	33	746.80 (+2)	1491.68
α.34	QQPNIAHASSQVSQQSY	33	937.28 (+2)	1871.88
α.35	QQPNIAHASSKVSQQSY	33	937.28 (+2)	1871.92
