# Supplementary material for: Isolation and characterization of gluten protein types from wheat, rye, barley and oats for use as reference materials
Source: PLoS One. 2017 Feb 24;12(2):e0172819. doi: 10.1371/journal.pone.0172819 (PMC5325591; doi:10.1371/journal.pone.0172819)
Supplement: S2 Table — Peptide sequences, their scores, m/z ratios, charge states and relative molecular weights (Mr). For corresponding protein sequences, see S6 Table. (PDF) [file pone.0172819.s005.pdf]

S2 Table. Peptides identified in each isolated rye gluten protein type. Peptide sequences, their scores, *m/z* ratios, charge states and relative molecular weights (M_r). For corresponding protein sequences, see S6 Table.

Abbre-viation	Gluten protein type<br>Peptide sequence	Peptide score	*m/z* (charge state)	M_r
	**HMW-secalins**			
HS.1	QQPAQGQQGYY	58	634.16 (+2)	1266.57
HS.2	KGGSFYPDETSPPQQL	56	875.79 (+2)	1749.82
HS.3	QQPGQGQQGY	55	545.65 (+2)	1089.50
HS.4	QKPGQGQQGY	53	545.63 (+2)	1089.52
HS.5	LQIGQGQQGY	52	546.21 (+2)	1090.55
HS.6	QQPGQGQQGYY	52	627.18 (+2)	1252.55
HS.7	QKPGQGQQGYY	52	627.18 (+2)	1252.59
HS.8	KRYYPSVTSPHQGSY	51	590.52 (+3)	1768.86
HS.9	ITSPQQSGQGQQPGQGQQPGQW	49	774.56 (+3)	2321.09
HG+HS.2	HVSAEHQAASL	48	575.11 (+2)	1148.56
HS.10	YITSPQQSGQGQQPGQGQQPGQW	47	828.82 (+3)	2484.15
HS.11	YPSVTSPHQGSY	46	661.74 (+2)	1321.60
HS.12	LTSPQQPGQGQQGYDSPY	46	975.80 (+2)	1949.88
HS.13	YSTSPRQPGQGQQEYYPTSPQQPGQW	46	999.12 (+3)	2994.36
HS+D	KVAKAQQL	43	443.18 (+2)	884.55
HS.14	YPTSPQQPGQGQQGY	41	818.23 (+2)	1634.74
HS.15	QQLQQGIFW	41	574.24 (+2)	1146.59
HS.16	QQPGQGQEGY	39	545.63 (+2)	1090.48
HS.17	QQPAQGQQGY	39	552.59 (+2)	1103.51
HS.18	YLTSPQQPGQGQQGY	39	826.28 (+2)	1650.77
HG+HS.3	HVSVEHQAASL	36	589.18 (+2)	1176.60
HS.19	QQPGQGQSGY	35	525.14 (+2)	1048.46
HS.20	QEPGQGQQGY	34	545.61 (+2)	1090.47
HS.21	LQIGQGQQGYYPTSPQQPGQGQQGY	33	903.60 (+3)	2707.27
HS.22	STSPRQPGQGQQEYYPTSPQQPGQW	31	1416.81 (+2)	2831.30
HS.23	KRYYPSVTSPHQGSYYPGQTSL	31	839.56 (+3)	2516.22
HS.24	STSPRQPGQGQQEY	30	781.66 (+2)	1561.72
HS.25	EQTTVPPKGGSFYPSETTPL	29	712.57 (+3)	2135.05
HG+HS.1	QQPGQGQQPGQW	28	669.71 (+2)	1337.62
HS.26	YPTSPQQPGQGQQPGQGQQGYY	28	1199.66 (+2)	2394.07
HS.27	LTSPQQPGQGQQGY	27	744.64 (+2)	1487.71
HS.28	STSLQQPGQGQQGHYPTSL	27	671.92 (+3)	2012.96
HS.29	YPSVTSPHQGSYYPGQTSL	26	1034.71 (+2)	2067.96
HS.30	KGGSFYPDETSPPQQLEQRIL	25	797.43 (+3)	2389.20
HS.31	QQPGQVQPGYY	18	632.69 (+2)	1263.60
	**γ-75k-secalins**			
γ75k.1	QQFPQQPQQPFPQQPL	68	968.26 (+2)	1934.97
γ75k.2	AQVQGIIQPQQL	61	661.83 (+2)	1321.74
γ75k.3	SQLEVVRSL	59	515.71 (+2)	1029.59
γ75k.4	QQHVGQGALAQVQGIIQPQQL	55	747.73 (+3)	2240.21
γ75k.5	ASIETGIVGH	52	492.17 (+2)	982.52
γ75k.6	ASIVTGIVGH	50	477.25 (+2)	952.54
γ75k.7	LPQSHKQHVGQGALAQVQGIIQPQQL	46	934.84 (+3)	2802.53
γ75k.8	QQHVGQGAL	45	469.21 (+2)	936.49
γ75k.9	LPQSHQQHVGQGALAQVQGIIQPQQL	40	934.84 (+3)	2802.50

γ75k.10	AQIPQQL	33	399.18 (+2)	796.45
γ75k.11	QQFPQQPQQSFPQQPQQPVPQQPL	24	943.73 (+3)	2827.41
	**ω-secalins**			
ωg+ωs.1	GQQPEQIISQRPQQPFPLQPQQQF	53	949.89 (+3)	2846.45
ωg+ωs.5	GQQPEQIISQRPQQPFPLQPKQPF	50	939.57 (+3)	2815.48
ωg+ωs.2	GEQPEQIISQRPQQPFPLQPQQPF	48	939.69 (+3)	2816.43
ωg+ωs.6	GQQPEQIISQRPQQPFPLQPQQPF	46	939.64 (+3)	2815.45
ωg+ωs.8	SLQPQQPVPQQPQRPF	44	625.60 (+3)	1873.99
ωg+ωs.7	GQQPMQIISQRPQQPFPLQPQQPF	43	939.58 (+3)	2817.45
ωg+ωs.3	AQQPEQIISQQPFPLQPQQPF	37	817.49 (+3)	2448.25
ωg+ωs.4	AQQPEQLISQQPFPLQPQQPF	35	1224.84 (+2)	2448.25
ωs.1	GQQPWQIISQRPQQPFPLQPQQPF	36	959.80 (+3)	2873.48
ωs.2	AQQPEQLISQQPFPL	36	862.85 (+2)	1722.90
ωs.3	RQLNPSEQEL	32	607.22 (+2)	1212.62
ωg+ωs.9	SQPQQPFPQQPGQIIPEQPQQPSPLQPQQPF	31	1173.89 (+3)	3518.77
ωs.4	ISQQPFPLQPQQPF	25	828.36 (+2)	1653.86
ωg+ωs.10	SQPQQPFPQQPGQIIPQQPQQPSPLQPQQPF	25	1173.97 (+3)	3517.78
ωg+ωs.12	GQQPEQIISRRPQQPFPLQPQQPF	21	949.89 (+3)	2843.49
ωg+ωs.11	SQPQQPFPQQPGQIIPKQPQQPSPLQPQQPF	17	1173.95 (+3)	3517.82
ωs.5	AQQPKQIISQQPFPLQPQQPF	17	1225.33 (+2)	2447.30
ωs.6	QSPQQPVPKEQSYPQQPYPSHQPFPTPQQY	17	1180.05 (+3)	3535.69
	**HMW-secalins within ω-secalins**			
HS.5	LQIGQGQQGY	56	546.21 (+2)	1090.55
HG+HS.2	HVSAEHQAASL	40	575.28 (+2)	1148.56
HS.12	LTSPQQPGQGQQGYDSPY	39	975.77 (+2)	1949.88
	**γ-40k-secalins**			
γ40k.1	FQIVQGQSIIQQQPAQLEVIRSL	75	1312.48 (+2)	2622.46
γ40k.2	FQIVQGQSIIQQQPAQL	70	963.82 (+2)	1925.08
γ40k.3	FKIVQGQSIIQQQPAQL	70	642.67 (+3)	1925.08
γ40k.4	FQIVQGKSIIQQQPAQL	70	642.67 (+3)	1925.08
γ40k.5	RPLFQIVQGQSIIQQQPAQL	66	765.32 (+3)	2291.28
γ40k.6	RPLFKIVQGQSIIQQQPAQL	66	1146.90 (+2)	2291.32
γ40k.7	RPLFQIVQGKSIIQQQPAQL	66	1146.90 (+2)	2291.32
γ40k.8	KIVQGQSIIQQQPAQL	60	889.82 (+2)	1778.01
γ40k.9	QIVQGQSIIQQQPAQL	60	889.83 (+2)	1777.98
γ40k.10	QIVQGKSIIQQQPAQL	60	889.82 (+2)	1778.01
γ40k.11	QIVQGQSIIQQQPAQLEVIRSL	52	826.31 (+3)	2475.39
γ40k.12	QIVQGQSIIQQQL	42	741.79 (+2)	1481.83
γ40k.13	VRPDCSNIRTPF	41	468.83 (+3)	1403.71
γ40k.14	IESSLQQQMNPCKNF	36	883.29 (+2)	1765.82
γ40k.15	IQSSLQQQMNPCKNF	23	883.23 (+2)	1764.84
γ40k.16	IKSSLQQQMNPCKNF	23	883.23 (+2)	1764.87
	**γ-75k-secalins within γ-40k- secalins**			
γ75k.3	SQLEVVRSL	51	515.71 (+2)	1029.59
γ75k.6	ASIVTGIVGH	45	477.17 (+2)	952.54
