# Supplementary material for: Isolation and characterization of gluten protein types from wheat, rye, barley and oats for use as reference materials
Source: PLoS One. 2017 Feb 24;12(2):e0172819. doi: 10.1371/journal.pone.0172819 (PMC5325591; doi:10.1371/journal.pone.0172819)
Supplement: S3 Table — Peptide sequences, their scores, m/z ratios, charge states and relative molecular weights (Mr). For corresponding protein sequences, see S7 Table. (PDF) [file pone.0172819.s006.pdf]

S3 Table. Peptides identified in each isolated barley gluten protein type. Peptide sequences, their scores, *m/z* ratios, charge states and relative molecular weights (M_r). For corresponding protein sequences, see S7 Table.

Abbre-viation	Gluten protein type<br>Peptide sequence	Peptide score	*m/z* (charge state)	M_r
	**D-hordeins**			
D.1	YYPDQTSSQQSW	69	745.15 (+2)	1488.62
D.2	LQPGQGQQGPY	61	586.70 (+2)	1171.57
D.3	TQQKPGQGYNPGGTSPL	55	863.26 (+2)	1728.85
HS+D	KVAKAQQL	46	443.12 (+2)	884.55
D.4	SQVVRQY	40	440.07 (+2)	878.47
D.5	QSATSPQQPGQGQGQQETYPIATSPHQPGQW	21	1107.38 (+3)	3318.54
D.6	QSATSPQQPGQGQGQQEPYPIATSPHQPGQW	19	1105.88 (+3)	3314.54
D.7	GGGLTTEQPQGGKQPF	17	801.17 (+2)	1600.79
D.8	SYPSATFPQQPGQGQGQQGYYPGATSL	17	938.98 (+3)	2814.30
	**C-hordeins**			
C.1	QPQQPFPQPQQPIAHQPQQPF	57	822.88 (+3)	2465.23
C.2	QPQQPFPQQPQQPLPRPQQPFPW	56	932.28 (+3)	2793.42
C.3	QPQQPFPQPQQPFPLQPQQPFPW	52	929.35 (+3)	2784.39
C.4	LQQPYPQNPY	49	624.21 (+2)	1246.61
C.5	IIPQQPQQPLPLQPHQPY	47	708.18 (+3)	2121.14
C.6	IIPQQPQQPFPLQPHQPY	41	719.37 (+3)	2155.13
C.7	SFSQQPQQPFPL	39	702.21 (+2)	1402.70
C.8	RQLNPSSQEL	39	586.17 (+2)	1170.61
C.9	LPQQPFPVQQPF	33	713.29 (+2)	1424.75
C.10	LPQKPFPVQQPF	33	713.29 (+2)	1424.79
C.11	SQQPQQPFPLQPQQPFPQQPQQPFPQQPQQIIF	25	1309.13 (+3)	3923.98
	**γ-hordeins within γ/B-hordeins**			
γh.1	VLPQQQAQF	50	529.69 (+2)	1057.56
γh.2	AQQQPSIEEQHQL	50	767.98 (+2)	1534.74
γh.3	TQQPYPQQQPL	40	664.12 (+2)	1326.66
γh.4	QPQQQPQFPQQKPF	22	863.24 (+2)	1724.87
	**B-hordeins within γ/B-hordeins**			
B.1	SIVLQEQPL	65	513.66 (+2)	1025.58
B.2	SIVLQEQSL	62	508.66 (+2)	1015.56
B.3	QQPQPQQLGQPQQVPQSVF	57	1081.65 (+2)	2161.10
B.4	QQPQPQQGQQQQVPQSVF	55	1040.67 (+2)	2079.02
B.5	SIVLQEQPQQL	52	641.62 (+2)	1281.70
B.6	LQEQQDQMLL	49	623.19 (+2)	1244.61
B.7	RIVPLAIDTRVGV	49	470.48 (+3)	1407.86
B.8	QQPQPQQVGQQQQVPQSAF	46	717.64 (+3)	2150.06
B.9	RGVGPSVGV	41	414.15 (+2)	826.47
B.10	RHEAVRAIVY	40	607.20 (+2)	1212.68
B.11	AIDTRVGV	39	415.59 (+2)	829.47
B.12	LQPHQLAQL	39	524.15 (+2)	1046.59
LG+B	LQPHQIAQL	39	524.15 (+2)	1046.59
B.13	VQVQIPF	33	415.64 (+2)	829.47
B.14	RHEAIRAIVY	31	614.23 (+2)	1226.70
B.15	GIDTRVGV	24	408.60 (+2)	815.46
B.16	LQLQIQY	22	904.77 (+1)	904.51

B.17	LQPHQIAQLEATTSIAL	19	917.59 (+2)	1833.01
B.18	RILRGVGPSVGV	17	605.25 (+2)	1208.74
B.19	FQQPQPQQLGQPQQVPQSVF	15	770.44 (+3)	2308.17
	**B-hordeins within B/γ-hordeins**			
B.2	SIVLQEQSL	69	508.66 (+2)	1015.56
B.4	QQPQPQQGQQQQVPQSVF	57	1040.32 (+2)	2079.02
B.7	RIVPLAIDTRVGV	51	470.22 (+3)	1407.86
B.1	SIVLQEQPL	50	513.67 (+2)	1025.58
B.3	QQPQPQQLGQPQQVPQSVF	47	1081.31 (+2)	2161.10
B.8	QQPQPQQVGQQQQVPQSAF	44	1076.23 (+2)	2150.06
B.10	RHEAVRAIVY	41	607.15 (+2)	1212.68
B.9	RGVGPSVGV	40	414.09 (+2)	826.47
B.11	AIDTRVGV	37	415.61 (+2)	829.47
B.14	RHEAIRAIVY	37	409.79 (+3)	1226.34
LG+B	LQPHQIAQL	33	524.17 (+2)	1046.59
B.13	VQVQIPF	33	415.64 (+2)	829.47
B.18	RILRGVGPSVGV	27	605.18 (+2)	1208.74
B.20	VLQQQCCQQLPQIPEQF	15	1014.30 (+2)	2028.98
	**γ-hordeins within B/γ-hordeins**			
γh.2	AQQQPSIEEQHQL	44	768.22 (+2)	1534.74
γh.3	TQQPYPQQQPL	20	665.16 (+2)	1326.66
