# Supplementary material for: Isolation and characterization of gluten protein types from wheat, rye, barley and oats for use as reference materials
Source: PLoS One. 2017 Feb 24;12(2):e0172819. doi: 10.1371/journal.pone.0172819 (PMC5325591; doi:10.1371/journal.pone.0172819)
Supplement: S4 Table — Peptide sequences, their scores, m/z ratios, charge states and relative molecular weights (Mr). For corresponding protein sequences, see S8 Table. (PDF) [file pone.0172819.s007.pdf]

S4 Table. Peptides identified in the oat avenin fraction. Peptide sequences, their scores, *m/z* ratios, charge states and relative molecular weights (M_r). For corresponding protein sequences, see S8 Table.

Abbre-viation	Gluten protein type<br>Peptide sequence	Peptide score	*m/z* (charge state)	M_r
	**avenins**			
A.1	LQPQLQQQIVQTQL	79	832.90 (+2)	1663.93
A.2	QQQQQQQQF	71	595.57 (+2)	1189.55
A.3	VQQQPPFVQQEQPF	69	850.43 (+2)	1698.84
A.4	QTQMQGQIEGMRAF	59	812.78 (+2)	1623.76
A.5	IQPQLQQQVF	58	613.70 (+2)	1227.67
A.6	VQQQQQQQQQQQPF	56	949.73 (+2)	1897.91
A.7	LQPQLQQQML	56	613.70 (+2)	1225.66
A.8	FQPQLQQQVF	54	631.73 (+2)	1261.65
A.9	QAQLQQQLL	52	535.24 (+2)	1068.60
A.10	LQPQVQQQL	51	541.16 (+2)	1080.60
A.11	LQPQLQQQL	50	548.20 (+2)	1094.62
A.12	NQPQQQAQF	47	544.64 (+2)	1087.51
A.13	QPQLQLQQQVF	47	678.73 (+2)	1355.73
A.14	QQQQQQQF	46	531.64 (+2)	1061.50
A.15	QQQAQVQQQVF	45	666.10 (+2)	1330.67
A.16	LQPQLQQQIL	44	604.60 (+2)	1207.70
A.17	FQPQMQQQF	44	591.10 (+2)	1180.54
A.18	QLQLQQQAQVQQQVF	44	907.21 (+2)	1812.96
A.19	NQPQMQGQIEGMRAF	44	867.87 (+2)	1733.81
A.20	QPQLQQQVF	43	558.19 (+2)	1114.58
A.21	VQQQQPF	41	437.58 (+2)	873.44
A.22	IQPQLQQVF	41	550.29 (+2)	1099.61
A.23	QPQQQAQF	39	487.62 (+2)	973.47
A.24	QQQLLQPQVQQQL	37	789.69 (+2)	1577.86
A.25	TTTVQYDPSEQYQPYPEQQEPF	37	892.20 (+3)	2674.18
A.26	VQQQQQQQPF	35	629.77 (+2)	1257.62
A.27	NPSEQYQPYPEQQEPF	35	991.14 (+2)	1979.86
A.28	QTQLQQQML	32	559.23 (+2)	1116.57
A.29	QKLQQQLL	31	499.53 (+2)	997.60
A.30	VQQQQMF	30	454.64 (+2)	907.43
A.31	QQQLIQPQLQQVF	27	799.36 (+2)	1596.87
A.32	FQPQMQQVTQGIF	27	776.68 (+2)	1550.76
A.33	QPPLQQQL	27	956.26 (+1)	950.53
A.34	DPSEQYQPYPEQQEPF	26	991.14 (+2)	1980.85
A.35	IPPQLQQVF	25	1069.41 (+1)	1068.60
A.36	LQPQVQQQLQQQL	22	789.13 (+2)	1577.86
A.37	TTTVQYNPSEQYQPYPEQQEPF	18	892.20 (+3)	2673.19
