# Supplementary material for: Isolation and characterization of gluten protein types from wheat, rye, barley and oats for use as reference materials
Source: PLoS One. 2017 Feb 24;12(2):e0172819. doi: 10.1371/journal.pone.0172819 (PMC5325591; doi:10.1371/journal.pone.0172819)
Supplement: S5 Table — The isolated wheat GPT were digested with chymotrypsin, analyzed by untargeted LC-MS/MS and the MS/MS files searched using the Mascot software and the NCBI Protein database (taxonomy Viridiplantae). (PDF) [file pone.0172819.s008.pdf]

S5 Table. Protein sequences (protein score > 63) identified in each isolated wheat gluten protein type (GPT). The isolated wheat GPT were digested with chymotrypsin, analyzed by untargeted LC-MS/MS and the MS/MS files searched using the Mascot software and the NCBI Protein database (taxonomy *Viridiplantae*).

**Protein type (number of hits) NCBI Accession**	**Protein score**	**Protein name**	**Organism**	**Number of peptide sequences**
**HMW-GS (64)**				
AGH32931.1	646	High molecular weight glutenin subunit 1Dx	*T. spelta*	17
CAI72574.1	644	HMW subunit of glutenin 1Dx2.2*	*T. aestivum*	17
AFP58009.1	644	High-molecular-weight glutenin 1Dx2.2	*T. aestivum*	17
AEO45112.2	643	High-molecular-weight glutenin subunit	*T. aestivum*	17
BAG12019.2	642	High-molecular-weight glutenin subunit 2.6	*T. aestivum*	17
AHN66476.1	642	High molecular weight glutenin subunit Dx5	*T. aestivum*	17
ABX89297.1	610	High molecular weight glutenin subunit	*T. aestivum*	16
AGH32939.1	610	High molecular weight glutenin subunit 1Dx	*Ae. tauschii*	16
AGH32941.1	610	High molecular weight glutenin subunit 1Dx	*T. spelta*	16
ABF14403.1	604	1Dx high molecular weight glutenin subunit	*Th. ponticum × T. aestivum*	16
BAH37041.1	601	High-molecular-weight glutenin subunit x5	*T. aestivum*	16
P10388.5	601	Glutenin, high molecular weight subunit DX5	*T. aestivum*	16
AGH32922.1	601	High molecular weight glutenin subunit 1Dx	*T. spelta*	16
AGH32923.1	601	High molecular weight glutenin subunit 1Dx	*T. spelta*	16
AGH32924.1	601	High molecular weight glutenin subunit 1Dx	*T. spelta*	16
AGH32925.1	601	High molecular weight glutenin subunit 1Dx	*T. spelta*	16
AGH32926.1	601	High molecular weight glutenin subunit 1Dx	*T. spelta*	16
AGH32927.1	601	High molecular weight glutenin subunit 1Dx	*T. spelta*	16
AGH32928.1	600	High molecular weight glutenin subunit 1Dx	*T. spelta*	16
AGH32929.1	600	High molecular weight glutenin subunit 1Dx	*T. spelta*	16
ADY38693.1	353	High-molecular-weight glutenin subunit	*S. cereale × T. aestivum*	8
ADY38694.1	353	High-molecular-weight glutenin subunit	*S. cereale × T. aestivum*	8

ADY38695.1	353	High-molecular-weight glutenin subunit	*S. cereale × T. aestivum*	8
ADY38712.1	353	High-molecular-weight glutenin subunit	*S. cereale × T. aestivum*	8
AEO19857.1	352	High-molecular-weight glutenin subunit	*T. aestivum*	8
ADY38698.1	351	High-molecular-weight glutenin subunit	*S. cereale × T. aestivum*	8
ADY38699.1	351	High-molecular-weight glutenin subunit	*S. cereale × T. aestivum*	8
ADY38717.1	351	High-molecular-weight glutenin subunit	*S. cereale × T. aestivum*	8
CAA43331.1	350	High-molecular-weight glutenin subunit 1Ax1	*T. aestivum*	8
ADY38692.1	350	High-molecular-weight glutenin subunit	*S. cereale × T. aestivum*	8
AHZ62762.1	350	High-molecular-weight glutenin subunit 1Ax1	*T. aestivum*	8
ADY38715.1	349	High-molecular-weight glutenin subunit	*S. cereale × T. aestivum*	8
CAA43361.1	293	HMW glutenin subunit 1By9	*T. aestivum*	9
AGZ95069.1	293	High molecular weight glutenin subunit By18	*T. aestivum*	9
AHI62991.1	293	High molecular weight glutenin subunit	*T. aestivum*	9
CAC40687.1	290	High molecular weight glutenin subunit y	*T. aestivum*	9
AAO64642.1	290	HMW glutenin subunit	*T. turgidum ssp. durum*	9
ABG68035.1	290	Y-type HMW glutenin	*T. aestivum*	9
AAQ93633.1	283	HMW-glutenin By subunit	*T. turgidum*	9
AHC72163.1	280	High molecular weight glutenin subunit 1By protein	*T. dicoccoides*	9
ABF82252.1	169	High molecular weight glutenin subunit	*T. aestivum*	4
BAN82580.1	169	High-molecular-weight glutenin subunit	*T. aestivum*	4
ACJ76973.1	164	High molecular weight glutenin x-type	*T. monococcum ssp. aegilopoides*	4
AEK20782.1	164	High molecular weight glutenin 1Ax2.1	*T. monococcum ssp. monococcum*	4
ACJ76970.1	164	High molecular weight glutenin x-type	*T. monococcum*	4
ABK54365.1	147	High molecular weight glutenin subunit	*T. aestivum*	3
AAU04841.1	139	High molecular weight glutenin subunit 1Dy10.1	*T. aestivum*	6
ABO86195.1	139	Dy-type high molecular weight subunit protein	*T. aestivum*	6
P10387.1	138	Glutenin, high molecular weight subunit DY10	*T. aestivum*	6

P08488.1	138	Glutenin, high molecular weight subunit 12	*T. aestivum*	6
AAR29050.1	138	HMW glutenin subunit	*T. aestivum*	6
AAR29051.1	138	HMW glutenin subunit	*T. aestivum*	6
ABF14404.1	138	1Dy high molecular weight glutenin subunit	*Th. ponticum* × *T. aestivum*	6
ABX64443.1	138	High molecular weight glutenin subunit	*T. aestivum*	6
BAF96643.1	138	High-molecular-weight glutenin subunit y10	*T. aestivum*	6
ACO56367.1	138	HMW glutenin subunit	*T. aestivum*	6
AHC72161.1	138	High molecular weight glutenin subunit 1Dy protein	*T. aestivum*	6
CAA32115.1	135	Unnamed protein product	*T. aestivum*	3
ABF14402.1	135	1Bx high molecular weight glutenin subunit	*T. aestivum*	3
BAE96560.1	135	High-molecular-weight glutenin subunit Bx17	*T. aestivum*	3
ABX89298.1	135	High molecular weight glutenin subunit	*T. aestivum*	3
AHC72166.1	135	High molecular weight glutenin subunit 1Bx protein	*T. aestivum* × *T. dicoccoides*	3
AAQ93629.1	118	HMW-glutenin Bx subunit	*T. turgidum*	3
AID62092.2	117	HMW-GS protein	*T. dicoccoides*	3
**ω5-gliadins (12)**				
AII26682.1	91	Fast omega-gliadin	*T. monococcum*	3
AII26683.1	91	Fast omega-gliadin	*T. monococcum*	3
AIU64833.1	91	Omega gliadin	*T. monococcum*	3
AIU64834.1	91	Omega gliadin	*T. monococcum*	3
AIU64840.1	91	Omega gliadin	*T. monococcum*	3
BAE20328.1	67	Omega-5 gliadin	*T. aestivum*	2
AJG03078.1	67	Omega-gliadin	*T. aestivum*	2
AJG03093.1	67	Omega-gliadin	*T. aestivum*	2
AJG03094.1	67	Omega-gliadin	*T. aestivum*	2
AKA88509.1	67	Fast omega-gliadin	*T. dicoccoides*	2
AKA88506.1	64	Fast omega-gliadin	*T. dicoccoides*	1
AKA88508.1	64	Fast omega-gliadin	*T. dicoccoides*	1
**ω1,2-gliadins (25)**				
BAN29067.1	162	Omega-gliadin, partial	*T. aestivum*	4
ACQ83639.1	106	Omega secalin	*T. aestivum*	3
ACN96903.1	102	Putative omega secalin	*T. aestivum*	3
ACN62213.1	98	Omega-gliadin, partial	*T. aestivum*	3
ACQ83634.1	96	Omega secalin	*S. cereale* × *T. aestivum*	3
ACQ83640.1	96	Omega secalin	*T. aestivum*	3
ACQ83637.1	96	Omega secalin	*T. aestivum*	3
ACQ83635.1	96	Omega secalin	*S. cereale* × *T. aestivum*	3
ACQ83642.1	95	Omega secalin	*T. aestivum*	3
CAA42836.1	94	Sec1 precursor	*S. cereale*	3
ACQ83629.1	94	Omega secalin	*S. cereale* × *T. turgidum*	3

ACQ83631.1	94	Omega secalin	*S. cereale × T. turgidum ssp. durum*	3
ACN62214.1	94	Omega-gliadin, partial	*T. aestivum*	3
ACQ83638.1	93	Omega secalin	*T. aestivum*	3
ACN96900.1	93	Putative omega secalin	*T. aestivum*	3
ACQ83626.1	89	Omega secalin	*S. cereale*	3
ADF58078.1	86	Omega-gliadin, partial	*T. urartu*	3
ADF58069.1	81	Omega-gliadin, partial	*T. monococcum*	3
ADF58072.1	81	Omega-gliadin, partial	*T. monococcum*	3
AEY70387.1	81	Omega-gliadin, partial	*Ae. tauschii*	3
ACN62216.1	79	Omega-gliadin, partial	*T. aestivum × L. elongatum*	3
ACQ83630.1	77	Omega secalin	*S. cereale × T. turgidum ssp. durum*	2
ACQ83633.1	76	Omega secalin	*S. cereale × T. aestivum*	3
ACO40286.1	68	Putative omega secalin	*T. aestivum*	2
ADF58077.1	67	Omega-gliadin, partial	*T. urartu*	2
**α-gliadins (63)**				
ACJ76939.1	224	Alfa gliadin	*T. monococcum*	5
AHN85627.1	224	Alpha-gliadin protein	*T. aestivum*	5
AHY37819.1	165	Alpha-gliadin	*T. aestivum*	4
ADA83689.1	157	Alpha-gliadin	*T. turgidum ssp. durum*	4
BAA12318.1	157	Alpha-gliadin	*T. aestivum*	4
AAZ73730.1	155	Alpha-type gliadin, partial	*T. dicoccoides*	4
CAB76955.1	147	Alpha-gliadin	*T. aestivum*	4
AFK32698.1	143	Alpha-gliadin protein	*Ae. tauschii*	6
BAM08452.1	139	Alpha/beta-gliadin	*T. aestivum*	4
BAM08455.1	139	Alpha/beta-gliadin	*T. aestivum*	4
BAM08456.1	139	Alpha/beta-gliadin	*T. aestivum*	4
BAM08458.1	139	Alpha/beta-gliadin	*T. aestivum*	4
AHY37815.1	139	Alpha-gliadin	*T. aestivum*	4
BAM08454.1	137	Alpha/beta-gliadin	*T. aestivum*	4
ABB92633.1	132	Alpha-gliadin protein	*T. turgidum ssp. durum*	3
AKB95618.1	129	Alpha-gliadin protein	*T. urartu*	3
AFQ13475.1	123	Alpha-gliadin	*T. aestivum*	3
AGO17679.1	120	Alpha-gliadin	*T. aestivum*	3
ABQ52119.1	118	Alpha-gliadin	*T. aestivum*	3
AFK32701.1	117	Alpha-gliadin protein	*Ae. tauschii*	4
AFK32696.1	117	Alpha-gliadin protein	*Ae. tauschii*	4
AAY45943.1	117	Alpha-gliadin storage protein	*Ae. tauschii*	3
AGW80506.1	117	Alpha-gliadin	*Ae. tauschii*	3
AAA96276.1	116	Alpha-gliadin	*T. aestivum*	3
CAB76962.1	109	Alpha-gliadin	*T. aestivum*	3
CAB76964.1	108	Alpha-gliadin	*T. aestivum*	3
AFQ13466.1	103	Alpha-gliadin	*T. aestivum*	3
AFQ13471.1	103	Alpha-gliadin	*T. aestivum*	3
AFX69619.1	103	Alpha-gliadin	*T. aestivum*	3

CAB76956.1	103	Alpha-gliadin	*T. aestivum*	3
AAZ94420.1	101	Alpha-/beta-gliadin storage protein precursor	*T. aestivum*	3
AFH74441.1	93	Alpha-gliadin	*T. dicoccoides*	3
AAZ94421.1	91	Alpha-/beta-gliadin storage protein precursor	*T. aestivum*	2
AAY45938.1	75	Alpha-gliadin storage protein	*Ae. tauschii*	2
ABS72145.1	71	Alpha gliadin	*T. aestivum*	2
ABS72157.1	71	Alpha gliadin	*Th. ponticum × T. aestivum*	2
AFX69609.1	71	Alpha-gliadin	*T. aestivum*	2
AFX69625.1	71	Alpha-gliadin	*T. aestivum*	2
AGO17644.1	71	Alpha-gliadin	*Ae. tauschii*	2
AGO17647.1	71	Alpha-gliadin	*Ae. tauschii*	2
AGW80509.1	71	Alpha-gliadin	*Ae. tauschii*	2
AHJ60676.1	71	Alpha-gliadin	*Ae. tauschii*	2
AAY45936.1	71	Alpha-gliadin storage protein, partial	*Ae. tauschii*	2
AAY45940.1	71	Alpha-gliadin storage protein, partial	*Ae. tauschii*	2
ADM96153.1	71	Alpha-gliadin storage protein	*Ae. tauschii*	2
ADM96157.1	71	Alpha-gliadin storage protein	*Ae. tauschii*	2
ADM96162.1	71	Alpha-gliadin storage protein	*Ae. tauschii*	2
AFK32693.1	71	Alpha-gliadin protein	*Ae. tauschii*	2
AFK32704.1	71	Alpha-gliadin protein	*Ae. tauschii*	2
AFK32706.1	71	Alpha-gliadin protein	*Ae. tauschii*	2
AFQ13463.1	70	Alpha-gliadin	*T. aestivum*	2
AFX69579.1	70	Alpha-gliadin	*T. aestivum*	2
AFX69600.1	70	Alpha-gliadin	*Ae. tauschii*	2
AGO17643.1	70	Alpha-gliadin	*Ae. tauschii*	2
AGO17645.1	70	Alpha-gliadin	*Ae. tauschii*	2
AGO17654.1	70	Alpha-gliadin	*Ae. tauschii*	2
AGO17675.1	70	Alpha-gliadin	*T. aestivum*	2
AGO17688.1	70	Alpha-gliadin	*T. aestivum*	2
AGW80492.1	70	Alpha-gliadin	*Ae. tauschii*	2
AGO17667.1	70	Alpha-gliadin	*T. aestivum*	2
ABQ52111.1	66	Alpha-gliadin	*Ae. tauschii*	2
AFX69616.1	66	Alpha-gliadin	*T. aestivum*	2
AGO17662.1	66	Alpha-gliadin	*T. aestivum*	2
**LMW-GS within α-gliadins (5)**				
ACA63873.1	134	Low molecular weight glutenin subunit	*T. aestivum*	4
ACA63856.1	132	Low molecular weight glutenin subunit	*T. aestivum*	4
ACA63857.1	132	Low molecular weight glutenin subunit	*T. aestivum*	4
AFI81534.1	132	Low molecular weight glutenin subunit, partial	*T. aestivum*	4
AFI81553.1	132	Low molecular weight glutenin subunit, partial	*T. aestivum*	4
**γ-gliadins (28)**				
AFX69682.1	176	Gamma-gliadin	*T. aestivum*	4
ACJ03494.1	174	Gamma-gliadin	*T. monococcum*	4
AFX69671.1	174	Gamma-gliadin	*T. monococcum*	4

AFX69673.1	174	Gamma-gliadin	*T. monococcum*	4
AFX69674.1	174	Gamma-gliadin	*T. monococcum*	4
AFX69675.1	174	Gamma-gliadin	*T. monococcum*	4
AGZ20271.1	174	Gamma-gliadin	*T. aestivum*	4
ACF93462.1	173	Gamma-gliadin	*T. aestivum*	4
AFX69679.1	173	Gamma-gliadin	*T. aestivum*	4
AFX69680.1	173	Gamma-gliadin	*T. aestivum*	4
AFX69681.1	173	Gamma-gliadin	*T. aestivum*	4
AGO17691.1	173	Gamma-gliadin	*T. aestivum*	4
AGO17692.1	173	Gamma-gliadin	*T. aestivum*	4
AGO17693.1	173	Gamma-gliadin	*T. aestivum*	4
AGO17696.1	173	Gamma-gliadin	*T. aestivum*	4
AGO17701.1	173	Gamma-gliadin	*T. aestivum*	4
ACJ03441.1	158	Gamma-gliadin	*T. turgidum*	4
ACJ03470.1	158	Gamma-gliadin	*T. aestivum*	4
P21292.1	158	RecName: Full=Gamma-gliadin; Flags: Precursor	*T. aestivum*	4
AEW46783.1	138	Gamma prolamin	*Ae. uniaristata*	3
AED99849.1	133	Gamma-gliadin	*T. monococcum*	3
AFC98435.1	133	Gamma-gliadin	*T. aestivum*	3
AFC98436.1	133	Gamma-gliadin	*T. aestivum*	3
AFC98437.1	133	Gamma-gliadin	*T. aestivum*	3
AFC98438.1	133	Gamma-gliadin	*T. aestivum*	3
AAF42989.1	130	Gamma-gliadin	*T. aestivum*	3
AFX69682.1	94	Gamma-gliadin	*T. aestivum*	3
ACI04100.1	84	Gamma-gliadin	*T. aestivum × L. elongatum*	2
**LMW-GS within γ-gliadins (9)**				
ACA63873.1	136	Low molecular weight glutenin subunit	*T. aestivum*	3
AGK83148.1	136	Low-molecular-weight glutenin subunit, partial	*T. aestivum*	3
AGK83270.1	136	Low-molecular-weight glutenin subunit, partial	*T. aestivum*	3
ACA63856.1	134	Low molecular weight glutenin subunit	*T. aestivum*	3
ACA63857.1	134	Low molecular weight glutenin subunit	*T. aestivum*	3
BAJ09388.1	134	Low molecular glutenin subunit	*T. aestivum*	3
AFI81534.1	134	Low molecular weight glutenin subunit, partial	*T. aestivum*	3
AFI81553.1	134	Low molecular weight glutenin subunit, partial	*T. aestivum*	3
AGK83348.1	134	Low-molecular-weight glutenin subunit, partial	*T. aestivum*	3
**LMW-GS (82)**				
AGK83348.1	368	Low-molecular-weight glutenin subunit, partial	*T. aestivum*	9
ACA63873.1	365	Low molecular weight glutenin subunit	*T. aestivum*	9
AGK83148.1	365	Low-molecular-weight glutenin subunit, partial	*T. aestivum*	9

AGK83270.1	365	Low-molecular-weight glutenin subunit, partial	*T. aestivum*	9
ACA63856.1	364	Low molecular weight glutenin subunit	*T. aestivum*	9
ACA63857.1	364	Low molecular weight glutenin subunit	*T. aestivum*	9
BAJ09388.1	364	Low molecular glutenin subunit	*T. aestivum*	9
AFI81534.1	364	Low molecular weight glutenin subunit, partial	*T. aestivum*	9
AFI81553.1	364	Low molecular weight glutenin subunit, partial	*T. aestivum*	9
ACC60296.1	303	Low molecular weight glutenin subunit	*Ae. uniaristata*	7
ACB98718.1	301	Low molecular weight glutenin subunit M3	*Ae. comosa*	7
AGO17755.1	273	LMW-GS, partial	*T. aestivum*	7
BAB78763.1	271	Low-molecular-weight glutenin subunit group 12 type VI	*T. aestivum*	7
BAB78764.1	271	Low-molecular-weight glutenin subunit group 12 type VI	*T. aestivum*	7
ACT98430.1	271	Low-molecular-weight glutenin subunit	*T. aestivum*	7
AGK83149.1	271	Low-molecular-weight glutenin subunit, partial	*T. aestivum*	7
AGK83250.1	271	Low-molecular-weight glutenin subunit, partial	*T. aestivum*	7
AGK83329.1	271	Low-molecular-weight glutenin subunit, partial	*T. aestivum*	7
AGO17737.1	271	LMW-GS, partial	*T. aestivum*	7
AGO17739.1	271	LMW-GS, partial	*T. aestivum*	7
AGO17734.1	270	LMW-GS, partial	*T. aestivum*	7
AAS10190.1	267	Low molecular weight glutenin	*T. aestivum*	6
ACT98424.1	267	Low-molecular-weight glutenin subunit	*T. aestivum*	6
AGK83145.1	267	Low-molecular-weight glutenin subunit, partial	*T. aestivum*	6
BAB78762.1	260	Low-molecular-weight glutenin subunit group 11 type VI	*T. aestivum*	6
ACP27640.1	259	Low molecular weight glutenin	*T. aestivum*	6
ACF93464.1	249	LMW-glutenin	*T. aestivum*	6
ABD72601.1	244	Low-molecular-weight glutenin subunit	*T. aestivum*	5
AGK83196.1	241	Low-molecular-weight glutenin subunit	*T. aestivum*	6
AEI00684.1	240	Low-molecular-weight glutenin subunit	*T. aestivum*	6
EMS57120.1	234	Glutenin, low molecular weight subunit	*T. urartu*	6
AIR77130.1	234	Low-molecular-weight glutenin subunit, partial	*T. urartu*	6
BAA23162.2	233	LMM glutenin 3	*T. aestivum*	6

ABB17936.1	223	Gamma-gliadin/LMW-glutenin chimera Ch2 precursor, partial	*T. aestivum*	6
ACJ76962.1	212	Low molecular weight glutenin	*T. monococcum*	5
ACJ76964.1	212	Low molecular weight glutenin	*T. monococcum*	5
ABQ50899.1	211	Type I LMW-glutenin LMW-i1	*T. monococcum*	5
AGU91691.1	207	LMW-i glutenin subunit 37	*T. aestivum*	5
ACF93465.1	207	LMW-glutenin	*T. aestivum*	5
ABY40381.1	206	Low-molecular-weight glutenin subunit	*Ae. longissima*	6
ACV32584.1	206	Low molecular weight glutenin	*Ae. speltoides*	6
AAP44991.1	197	Low molecular weight glutenin precursor	*T. aestivum ssp. tibeticum*	4
ACX46117.1	197	Low molecular weight glutenin subunit m1	*T. timopheevii ssp. armeniacum*	4
AAB48475.1	195	Low-molecular-weight glutenin storage protein	*T. aestivum*	4
BAB78759.1	195	Low-molecular-weight glutenin subunit group 8 type IV	*T. aestivum*	4
BAB78760.1	195	Low-molecular-weight glutenin subunit group 9 type IV	*T. aestivum*	4
AAU04842.1	195	Low molecular weight glutenin subunit XJLMW-1347	*T. aestivum*	4
AAV75994.1	195	Low molecular weight glutenin subunit	*Th. ponticum × T. aestivum*	4
AAV92011.1	195	Low molecular weight glutenin	*T. aestivum*	4
ABG76007.1	195	Low molecular weight glutenin subunit GF-2	*T. aestivum*	4
ABV03149.1	195	Low molecular weight glutenin, partial	*T. turgidum*	4
ABV72239.1	195	Low molecular weight glutenin subunit	*Th. ponticum × T. aestivum*	4
ABV72242.1	195	Low molecular weight glutenin subunit	*Th. ponticum × T. aestivum*	4
ABV72243.1	195	Low molecular weight glutenin subunit	*Th. ponticum × T. aestivum*	4
ABV72244.1	195	Low molecular weight glutenin subunit	*Th. ponticum × T. aestivum*	4
ABV72248.1	195	Low molecular weight glutenin subunit	*Th. ponticum × T. aestivum*	4
ABV72249.1	195	Low molecular weight glutenin subunit	*Th. ponticum × T. aestivum*	4
ABV72253.1	195	Low molecular weight glutenin subunit	*Th. ponticum × T. aestivum*	4
ABZ79361.1	195	Low molecular weight glutenin subunit	*T. timopheevii*	4
ABZ79362.1	195	Low molecular weight glutenin subunit	*T. timopheevii*	4
AEO21443.1	195	Low molecular weight glutenin subunit, partial	*T. aestivum*	4
ABY58128.1	195	LMW-m glutenin subunit 0072P19-M	*T. aestivum*	4

ABV72238.1	195	Low molecular weight glutenin subunit	*Th. ponticum × T. aestivum*	4
BAA22613.2	193	LMM glutenin 1	*T. aestivum*	4
AAP44992.1	191	Low molecular weight glutenin precursor	*T. aestivum ssp. tibeticum*	4
ADH51279.1	182	LMW glutenin subunit Glu-D3	*T. aestivum*	4
CAA74550.1	181	Low molecular weight glutenin subunit	*T. turgidum ssp. durum*	4
AAT36483.1	172	s-Type low molecular weight glutenin L4-36	*T. aestivum*	5
AAT37861.1	172	Low molecular weight glutenin subunit LMW.S6 precursor	*Ae. tauschii*	5
ABI21861.1	172	Low molecular weight glutenin	*T. aestivum*	5
ABY58134.1	172	LMW-m glutenin subunit 0703A9-M	*T. aestivum*	5
ACY08820.1	172	Low molecular weight glutenin subunit D3-1	*T. aestivum*	5
AEI00697.1	172	Low-molecular-weight glutenin subunit	*T. aestivum*	5
AGK83315.1	172	Low-molecular-weight glutenin subunit	*T. aestivum*	5
AGK83315.1	172	Low-molecular-weight glutenin subunit	*T. aestivum*	5
AEI00683.1	172	Low-molecular-weight glutenin subunit	*T. aestivum*	5
AAX98173.1	157	Low molecular weight glutenin subunit	*T. aestivum*	3
CAB40553.1	148	Low molecular weight glutenin subunit	*T. turgidum ssp. durum*	4
AFV79559.1	146	Low molecular weight glutenin subunit	*T. dicoccoides*	3
CAC05403.1	145	Low molecular weight glutenin subunit	*T. turgidum ssp. durum*	3
AAT76905.1	145	Low molecular weight protein	*Ae. tauschii × T. turgidum*	3
AGK83307.1	145	Low-molecular-weight glutenin subunit, partial	*T. aestivum*	3
**α-gliadins within LMW-GS (3)**				
BAM08455.1	231	Alpha/beta-gliadin	*T. aestivum*	5
BAM08452.1	153	Alpha/beta-gliadin	*T. aestivum*	4
BAM08454.1	153	Alpha/beta-gliadin	*T. aestivum*	4
