# Supplementary material for: Isolation and characterization of gluten protein types from wheat, rye, barley and oats for use as reference materials
Source: PLoS One. 2017 Feb 24;12(2):e0172819. doi: 10.1371/journal.pone.0172819 (PMC5325591; doi:10.1371/journal.pone.0172819)
Supplement: S6 Table — The isolated rye GPT were digested with chymotrypsin, analyzed by untargeted LC-MS/MS and the MS/MS files searched using the Mascot software and the NCBI Protein database (taxonomy Viridiplantae). (PDF) [file pone.0172819.s009.pdf]

S6 Table. Protein sequences (protein score > 63) identified in each isolated rye gluten protein type (GPT). The isolated rye GPT were digested with chymotrypsin, analyzed by untargeted LC-MS/MS and the MS/MS files searched using the Mascot software and the NCBI Protein database (taxonomy *Viridiplantae*).

Protein type (number of hits) NCBI Accession	Protein score	Protein name	Organism	Number of peptide sequences
**HMW-secalins (14)**				
CAC40670.1	670	High molecular weight glutenin subunit x	*S. cereale ssp. segetale*	16
CAC40674.1	670	High molecular weight glutenin subunit x	*S. cereale ssp. ancestrale*	16
CAC40680.1	670	High molecular weight glutenin subunit x	*S. cereale*	16
ADC79689.1	670	HMW glutenin subunit Rx	*S. cereale*	16
AHI62993.1	669	High molecular weight glutenin subunit x	*T. aestivum*	16
CAC40677.1	283	High molecular weight glutenin subunit x	*S. cereale ssp. dighoricum*	6
CAC40671.1	281	High molecular weight glutenin subunit x	*S. cereale ssp. afghanicum*	6
CAC40672.1	128	High molecular weight glutenin subunit y	*S. cereale ssp. afghanicum*	4
AGS18765.1	109	High-molecular-weight glutenin subunit	*S. cereale × T. aestivum*	3
AAF23507.1	81	Glutenin, high molecular weight subunit type y precursor	*T. aestivum*	2
CAC40669.1	81	High molecular weight glutenin subunit y	*S. cereale*	2
CAC40676.1	81	High molecular weight glutenin subunit y	*S. cereale ssp. dighoricum*	2
CAC40681.1	81	High molecular weight glutenin subunit y	*S. cereale*	2
CAC40682.1	81	High molecular weight glutenin subunit y	*S. cereale*	2
**γ-75k-secalins (65)**				
ABO32295.1	270	75k gamma secalin	*S. vavilovii*	5
AFX60464.1	270	75k gamma secalin	*S. cereale*	5
AEZ06411.1	267	75k gamma secalin	*S. strictum*	5
AAG35598.1	265	Secalin precursor	*S. cereale*	5
ADP95481.1	265	75k gamma secalin	*S. cereale*	5
AEZ06410.1	265	75k gamma secalin	*S. strictum*	5
AFX60462.1	233	75k gamma secalin	*S. cereale*	5
AFX60463.1	233	75k gamma secalin	*S. cereale*	5
AFX60467.1	233	75k gamma secalin	*S. cereale*	5
ADP95480.1	229	75k gamma secalin	*S. cereale*	5
AEZ06409.1	229	75k gamma secalin	*S. strictum*	5
ADP95479.1	227	75k gamma secalin	*S. cereale*	5
ADP95517.1	227	75k gamma secalin	*T. aestivum*	5

AFX60458.1	221	75k gamma secalin	*S. strictum ssp. africanum*	4
ADP95487.1	214	75k gamma secalin	*T. aestivum*	5
ADP95493.1	214	75k gamma secalin	*T. aestivum*	5
ADP95512.1	214	75k gamma secalin	*T. aestivum*	5
ADP95513.1	214	75k gamma secalin	*T. aestivum*	5
ADP95520.1	214	75k gamma secalin	*T. aestivum*	5
ABO32293.1	171	75k gamma secalin	*S. sylvestre*	3
ADP95484.1	171	75k gamma secalin	*S. cereale*	3
ADP95502.1	171	75k gamma secalin	*T. aestivum*	3
ADP95504.1	171	75k gamma secalin	*T. aestivum*	3
AFX60455.1	171	75k gamma secalin	*S. strictum ssp. africanum*	3
ABO32294.1	169	Secalin precursor	*S. cereale*	3
ADP95482.1	169	75k gamma secalin	*S. cereale*	3
ADP95483.1	169	75k gamma secalin	*S. cereale*	3
ADP95487.1	169	75k gamma secalin	*S. cereale*	3
ADP95490.1	169	75k gamma secalin	*S. cereale*	3
ADP95491.1	169	75k gamma secalin	*S. cereale*	3
ADP95492.1	169	75k gamma secalin	*T. aestivum*	3
ADP95494.1	169	75k gamma secalin	*T. aestivum*	3
ADP95495.1	169	75k gamma secalin	*T. aestivum*	3
ADP95496.1	169	75k gamma secalin	*T. aestivum*	3
ADP95499.1	169	75k gamma secalin	*T. aestivum*	3
ADP95501.1	169	75k gamma secalin	*T. aestivum*	3
ADP95503.1	169	75k gamma secalin	*T. aestivum*	3
ADP95505.1	169	75k gamma secalin	*T. aestivum*	3
ADP95506.1	169	75k gamma secalin	*T. aestivum*	3
ADP95507.1	169	75k gamma secalin	*T. aestivum*	3
ADP95508.1	169	75k gamma secalin	*T. aestivum*	3
ADP95510.1	169	75k gamma secalin	*T. aestivum*	3
ADP95514.1	169	75k gamma secalin	*T. aestivum*	3
ADP95515.1	169	75k gamma secalin	*T. aestivum*	3
ADP95516.1	169	75k gamma secalin	*T. aestivum*	3
ADP95519.1	169	75k gamma secalin	*T. aestivum*	3
ADP95521.1	169	75k gamma secalin	*T. aestivum*	3
ADP95522.1	169	75k gamma secalin	*T. aestivum*	3
ADP95524.1	169	75k gamma secalin	*T. aestivum*	3
ADP95525.1	169	75k gamma secalin	*T. aestivum*	3
ADP95526.1	169	75k gamma secalin	*T. aestivum*	3
ADP95527.1	169	75k gamma secalin	*T. aestivum*	3
ADP95528.1	169	75k gamma secalin	*T. aestivum*	3
ADP95529.1	169	75k gamma secalin	*T. aestivum*	3
ADP95530.1	169	75k gamma secalin	*T. aestivum*	3
AEZ06408.1	169	75k gamma secalin	*S. strictum*	3
AEZ06412.1	169	75k gamma secalin	*S. strictum*	3
AFX60451.1	169	75k gamma secalin	*S. strictum ssp. africanum × T. turgidum ssp. durum*	3

AFX60452.1	169	75k gamma secalin	*S. strictum ssp. africanum × T. turgidum ssp. durum*	3
AFX60453.1	169	75k gamma secalin	*S. strictum ssp. africanum × T. turgidum ssp. durum*	3
AFX60456.1	169	75k gamma secalin	*S. strictum ssp. africanum*	3
AFX60457.1	169	75k gamma secalin	*S. strictum ssp. africanum*	3
AFX60459.1	169	75k gamma secalin	*S. strictum ssp. africanum*	3
ADP95518.1	169	75k gamma secalin	*T. aestivum*	3
ADP95485.1	169	75k gamma secalin	*S. cereale*	3
**ω-secalins (16)**				
ACQ83642.1	128	Omega secalin	*T. aestivum*	4
ACQ83639.1	125	Omega secalin	*T. aestivum*	4
ACQ83635.1	125	Omega secalin	*S. cereale × T. aestivum*	4
CAA42836.1	125	Sec1 precursor	*S. cereale*	4
ACQ83629.1	125	Omega secalin	*S. cereale × T. turgidum ssp. durum*	4
ACQ83631.1	125	Omega secalin	*S. cereale × T. turgidum ssp. durum*	4
ACN96903.1	123	Putative omega secalin	*T. aestivum*	4
ACQ83628.1	123	Omega secalin	*S. cereale*	4
CAA42837.1	104	Sec1 precursor	*S. cereale*	4
ACQ83627.1	101	Omega secalin	*S. cereale*	3
ACO40286.1	101	Putative omega secalin	*T. aestivum*	3
ACO40284.1	91	Putative omega secalin	*T. aestivum*	2
ACN96900.1	89	Putative omega secalin	*T. aestivum*	2
ACQ83633.1	79	Omega secalin	*S. cereale × T. aestivum*	3
ACQ83625.1	67	Putative omega secalin	*T. aestivum*	2
AAB37407.1	65	omega 1-40 secalin isoform P1-2=coeliac immunoreactive protein/prolamin {N-terminal} [Secale cereale=rye, cv. Petkus, seed endosperm, Peptide Partial, 24 aa]	*S. cereale*	1
**HMW-secalins within ω-secalins (4)**				
CAC40670.1	130	High molecular weight glutenin subunit x	*S. cereale ssp. segetale*	3
CAC40674.1	130	High molecular weight glutenin subunit x	*S. cereale ssp. segetale*	3
CAC40680.1	130	High molecular weight glutenin subunit x	*S. cereale*	3
ADC79689.1	130	HMW glutenin subunit Rx	*S. cereale*	3

**γ-40k-secalins (4)**				
AEW46799.1	388	Gamma prolamin	*S. cereale ssp. afghanicum*	7
AEW46832.1	219	Gamma prolamin, partial	*S. cereale ssp. afghanicum*	4
AEW46838.1	219	Gamma prolamin, partial	*S. strictum ssp. anatolicum*	4
AEW46841.1	78	Gamma prolamin	*S. cereale*	2
**γ-75k-secalins within γ-40k- secalins (66)**				
AAG35598.1	96	Secalin precursor	*S. cereale*	2
ABO32293.1	96	75k gamma secalin	*S. sylvestre*	2
ADP95484.1	96	75k gamma secalin	*S. cereale*	2
AFX60455.1	96	75k gamma secalin	*S. strictum ssp. africanum*	2
ABO32294.1	96	Secalin precursor	*S. cereale*	2
ABO32295.1	96	75k gamma secalin	*S. vavilovii*	2
ABO32296.1	96	75k gamma secalin	*S. strictum*	2
ADP95479.1	96	75k gamma secalin	*S. cereale*	2
ADP95481.1	96	75k gamma secalin	*S. cereale*	2
ADP95482.1	96	75k gamma secalin	*S. cereale*	2
ADP95483.1	96	75k gamma secalin	*S. cereale*	2
ADP95485.1	96	75k gamma secalin	*S. cereale*	2
ADP95487.1	96	75k gamma secalin	*S. cereale*	2
ADP95488.1	96	75k gamma secalin	*T. aestivum*	2
ADP95489.1	96	75k gamma secalin	*T. aestivum*	2
ADP95490.1	96	75k gamma secalin	*S. cereale*	2
ADP95491.1	96	75k gamma secalin	*S. cereale*	2
ADP95492.1	96	75k gamma secalin	*T. aestivum*	2
ADP95494.1	96	75k gamma secalin	*T. aestivum*	2
ADP95495.1	96	75k gamma secalin	*T. aestivum*	2
ADP95496.1	96	75k gamma secalin	*T. aestivum*	2
ADP95497.1	96	75k gamma secalin	*T. aestivum*	2
ADP95498.1	96	75k gamma secalin	*T. aestivum*	2
ADP95499.1	96	75k gamma secalin	*T. aestivum*	2
ADP95500.1	96	75k gamma secalin	*T. aestivum*	2
ADP95501.1	96	75k gamma secalin	*T. aestivum*	2
ADP95502.1	96	75k gamma secalin	*T. aestivum*	2
ADP95503.1	96	75k gamma secalin	*T. aestivum*	2
ADP95504.1	96	75k gamma secalin	*T. aestivum*	2
ADP95505.1	96	75k gamma secalin	*T. aestivum*	2
ADP95506.1	96	75k gamma secalin	*T. aestivum*	2
ADP95507.1	96	75k gamma secalin	*T. aestivum*	2
ADP95508.1	96	75k gamma secalin	*T. aestivum*	2
ADP95510.1	96	75k gamma secalin	*T. aestivum*	2
ADP95514.1	96	75k gamma secalin	*T. aestivum*	2
ADP95515.1	96	75k gamma secalin	*T. aestivum*	2
ADP95516.1	96	75k gamma secalin	*T. aestivum*	2
ADP95519.1	96	75k gamma secalin	*T. aestivum*	2
ADP95521.1	96	75k gamma secalin	*T. aestivum*	2
ADP95522.1	96	75k gamma secalin	*T. aestivum*	2
ADP95524.1	96	75k gamma secalin	*T. aestivum*	2
ADP95525.1	96	75k gamma secalin	*T. aestivum*	2

ADP95526.1	96	75k gamma secalin	*T. aestivum*	2
ADP95527.1	96	75k gamma secalin	*T. aestivum*	2
ADP95528.1	96	75k gamma secalin	*T. aestivum*	2
ADP95529.1	96	75k gamma secalin	*T. aestivum*	2
ADP95530.1	96	75k gamma secalin	*T. aestivum*	2
AEZ06408.1	96	75k gamma secalin	*S. strictum*	2
AEZ06412.1	96	75k gamma secalin	*S. strictum*	2
AFX60442.1	96	75k gamma secalin	*S. strictum ssp. africanum*	2
AFX60443.1	96	75k gamma secalin	*S. strictum ssp. africanum*	2
AFX60446.1	96	75k gamma secalin	*S. strictum ssp. africanum*	2
AFX60447.1	96	75k gamma secalin	*S. strictum ssp. africanum*	2
AFX60450.1	96	75k gamma secalin	*S. strictum ssp. africanum*	2
AFX60451.1	96	75k gamma secalin	*S. strictum ssp. africanum × T. turgidum ssp. durum*	2
AFX60452.1	96	75k gamma secalin	*S. strictum ssp. africanum × T. turgidum ssp. durum*	2
AFX60453.1	96	75k gamma secalin	*S. strictum ssp. africanum × T. turgidum ssp. durum*	2
AFX60456.1	96	75k gamma secalin	*S. strictum ssp. africanum*	2
AFX60457.1	96	75k gamma secalin	*S. strictum ssp. africanum*	2
AFX60458.1	96	75k gamma secalin	*S. strictum ssp. africanum*	2
AFX60459.1	96	75k gamma secalin	*S. strictum ssp. africanum*	2
AFX60462.1	96	75k gamma secalin	*S. cereale*	2
AFX60463.1	96	75k gamma secalin	*S. cereale*	2
AFX60464.1	96	75k gamma secalin	*S. cereale*	2
AFX60467.1	96	75k gamma secalin	*S. cereale*	2
ADP95518.1	96	75k gamma secalin	*T. aestivum*	2
