# Supplementary material for: Isolation and characterization of gluten protein types from wheat, rye, barley and oats for use as reference materials
Source: PLoS One. 2017 Feb 24;12(2):e0172819. doi: 10.1371/journal.pone.0172819 (PMC5325591; doi:10.1371/journal.pone.0172819)
Supplement: S7 Table — The isolated barley GPT were digested with chymotrypsin, analyzed by untargeted LC-MS/MS and the MS/MS files searched using the Mascot software and the NCBI Protein database (taxonomy Viridiplantae). (PDF) [file pone.0172819.s010.pdf]

S7 Table. Protein sequences (protein score > 63) identified in each isolated barley gluten protein type (GPT). The isolated barley GPT were digested with chymotrypsin, analyzed by untargeted LC-MS/MS and the MS/MS files searched using the Mascot software and the NCBI Protein database (taxonomy *Viridiplantae*).

Protein type (number of hits) NCBI Accession	Protein score	Protein name	Organism	Number of peptide sequences
**D-hordeins (6)**				
BAA11642.1	318	D hordein	*H. vulgare ssp. vulgare*	8
BAK04239.1	318	Predicted protein	*H. vulgare ssp. vulgare*	8
AFM77735.1	318	D hordein	*H. vulgare*	8
AFM77736.1	318	D hordein	*H. vulgare*	8
AFM77750.1	318	D hordein	*H. vulgare ssp. vulgare*	8
AAP31051.1	224	D hordein	*H. vulgare*	6
**C-hordeins (6)**				
AAB28161.1	310	C-hordein	*H. vulgare*	7
CAA42642.1	183	Unnamed protein product	*H. vulgare ssp. vulgare*	4
AFM77749.1	143	C hordein	*H. vulgare ssp. vulgare*	3
P02864.1	87	RecName: Full=C-hordein	*H. vulgare ssp. spontaneum*	2
P17991.1	64	RecName: Full=C-hordein; AltName: Full=Clone PC HOR1-3	*H. vulgare*	2
AAA32942.1	64	C-hordein, partial	*H. vulgare*	2
**γ-hordeins within γ/B-hordeins (3)**				
CAA51204.1	160	Gamma 3 hordein, partial	*H. vulgare*	4
P80198.1	160	RecName: Full=Gamma-hordein-3	*H. vulgare*	4
AFM77739.1	160	Gamma 3 hordein	*H. vulgare*	4
**B-hordeins within γ/B-hordeins (23)**				
ABB82613.1	255	B hordein	*H. vulgare ssp. vulgare*	5
ACU09490.1	255	B hordein	*H. vulgare ssp. vulgare*	5
ACU09494.1	255	B hordein	*H. vulgare ssp. vulgare*	5
P06471.1	227	RecName: Full=B3-hordein	*H. vulgare*	5
AFM77744.1	223	B3 hordein	*H. vulgare*	5
AFM77745.1	223	B3 hordein	*H. vulgare*	5
ABH01262.1	193	B hordein	*H. vulgare ssp. vulgare*	4
ACU09491.1	167	B hordein	*H. vulgare ssp. vulgare*	4
P06470.1	147	RecName: Full=B1-hordein; Flags: Precursor	*H. vulgare*	4
CAA25913.1	147	Unnamed protein product, partial	*H. vulgare*	4

1103203A	147	Hordein B	*H. vulgare ssp. vulgare*	4
AFM77747.1	135	B3 hordein	*H. vulgare*	3
AFM77740.1	125	B1 hordein	*H. vulgare*	3
AFM77741.1	125	B1 hordein	*H. vulgare*	3
AFM77742.1	125	B1 hordein	*H. vulgare*	3
AFM77743.1	125	B1 hordein	*H. vulgare*	3
CAA25509.1	123	Unnamed protein product, partial	*H. vulgare*	3
CAA60681.1	123	B1 hordein	*H. vulgare*	3
AFM37566.1	123	B-hordein	*H. vulgare ssp. vulgare*	3
AFM77732.1	123	B-hordein	*H. vulgare ssp. vulgare*	3
AKZ20951.1	123	B-hordein precursor	*H. vulgare ssp. vulgare*	3
AAZ76368.1	82	B hordein	*H. vulgare ssp. vulgare*	2
AFM77746.1	79	B3 hordein	*H. vulgare*	2
**B-hordeins within B/γ-hordeins (17)**				
P06471.1	318	RecName: Full=B3-hordein	*H. vulgare*	7
AFM77744.1	249	B3 hordein	*H. vulgare*	6
ABB82613.1	229	B hordein	*H. vulgare ssp. vulgare*	5
ACU09490.1	229	B hordein	*H. vulgare ssp. vulgare*	5
P06470.1	177	RecName: Full=B1-hordein; Flags: Precursor	*H. vulgare*	5
CAA25913.1	177	Unnamed protein product, partial	*H. vulgare*	5
ABH01262.1	154	B hordein	*H. vulgare ssp. vulgare*	4
AFM77740.1	116	B1 hordein	*H. vulgare*	3
AFM77741.1	116	B1 hordein	*H. vulgare*	3
AFM77742.1	116	B1 hordein	*H. vulgare*	3
AFM77743.1	116	B1 hordein	*H. vulgare*	3
CAA60681.1	115	B1 hordein	*H. vulgare*	3
CAA25509.1	115	Unnamed protein product, partial	*H. vulgare*	3
AFM37566.1	115	B-hordein	*H. vulgare ssp. vulgare*	3
AFM77732.1	115	B-hordein	*H. vulgare ssp. vulgare*	3
AFM77747.1	112	B3 hordein	*H. vulgare*	3
AAZ76368.1	92	B hordein	*H. vulgare ssp. vulgare*	2
**γ-hordeins within B/γ-hordeins (3)**				
CAA51204.1	88	Gamma 3 hordein, partial	*H. vulgare*	4
P80198.1	88	RecName: Full=Gamma-hordein-3	*H. vulgare*	4
AFM77739.1	88	Gamma 3 hordein	*H. vulgare*	4
