# Supplementary material for: Isolation and characterization of gluten protein types from wheat, rye, barley and oats for use as reference materials
Source: PLoS One. 2017 Feb 24;12(2):e0172819. doi: 10.1371/journal.pone.0172819 (PMC5325591; doi:10.1371/journal.pone.0172819)
Supplement: S8 Table — The isolated avenin fraction was digested with chymotrypsin, analyzed by untargeted LC-MS/MS and the MS/MS files were searched using the Mascot software and the NCBI Protein database (taxonomy Viridiplantae). (PDF) [file pone.0172819.s011.pdf]

S8 Table. Protein sequences (protein score > 63) identified in oat avenins. The isolated avenin fraction was digested with chymotrypsin, analyzed by untargeted LC-MS/MS and the MS/MS files were searched using the Mascot software and the NCBI Protein database (taxonomy *Viridiplantae*).

**NCBI Accession**<br><br>**Avenins (49)**	**Protein score**	**Protein name**	**Organism**	**Number of peptide sequences**
CCH22485.1	407	Avenin	*A. eriantha*	9
CCH22488.1	405	Avenin	*A. murphyi*	9
CCH22489.1	404	Avenin	*A. magna*	9
CCH22518.1	380	Avenin	*A. prostrata*	9
CCH22504.1	379	Avenin	*A. canariensis*	9
CCH22487.1	378	Avenin	*A. longiglumis*	9
CCH22493.1	365	Avenin	*A. macrostachya*	9
CCH22492.1	362	Avenin, partial	*A. murphyi*	9
CCH22519.1	361	Avenin	*A. sativa*	8
CBL51494.1	359	Avenin protein, partial	*A. sativa*	8
CCH22520.1	358	Avenin	*A. strigosa*	8
CCH22490.1	354	Avenin	*A. ventricosa*	8
CCH22491.1	354	Avenin	*A. macrostachya*	8
CCC80643.1	337	Avenin protein, partial	*A. longiglumis*	7
CBL51496.1	279	Avenin protein, partial	*A. sativa*	6
AGB56857.1	277	Gliadin-like avenin	*A. sativa*	6
CBL51489.1	230	Avenin protein, partial	*A. sativa*	6
CCH22495.1	230	Avenin, partial	*A. sativa*	6
CCH22496.1	230	Avenin	*A. insularis*	6
AGB56872.1	225	Gliadin-like avenin	*A. sativa*	5
CCC80650.1	202	Avenin protein, partial	*A. prostrata*	5
Q09114.1	189	RecName: Full=Avenin-E; AltName: Full=Alpha-2 avenin; AltName: Full=Avenin N9; AltName: Full=Celiac immunoreactive protein 3; Short=CIP-3; AltName: Full=Prolamin	*A. sativa*	5
CCC80653.1	181	Avenin protein, partial	*A. strigosa*	4
AAA32716.1	181	Avenin	*A. sativa*	4
CCC80651.1	181	Avenin protein, partial	*A. longiglumis*	4
CCC80677.1	159	Avenin protein, partial	*A. macrostachya*	4
CCC80673.1	158	Avenin protein, partial	*A. murphyi*	4
AGB56858.1	158	Gliadin-like avenin	*A. sativa*	4
CBL51488.1	158	Avenin protein, partial	*A. sativa*	4
AGB56866.1	148	Gliadin-like avenin	*A. sativa*	3
AGB56870.1	148	Gliadin-like avenin	*A. sativa*	3
CCC80672.1	148	Avenin protein, partial	*A. murphyi*	3
CCC80644.1	148	Avenin protein, partial	*A. damascena*	3
CCC80640.1	148	Avenin protein, partial	*A. strigosa*	3
AGB56874.1	147	Gliadin-like avenin	*A. sativa*	3
CCC80676.1	129	Avenin protein, partial	*A. insularis*	3
CCC80647.1	129	Avenin protein, partial	*A. canariensis*	3
AGB56868.1	118	Gliadin-like avenin	*A. sativa*	3
CCC80661.1	98	Avenin protein, partial	*A. ventricosa*	3
CCC80659.1	98	Avenin protein, partial	*A. clauda*	3

Q09097.1	94	RecName: Full=Avenin-F; AltName: Full=Celiac immunoreactive protein 2; Short=CIP-2; AltName: Full=Gamma-3-avenin; AltName: Full=Prolamin	*A. sativa*	3
AAB32025.1	77	Alcohol-soluble avenin-3=23.2 kda protein [Avena sativa=oat, Narymsky 943, Peptide, 201 aa]	*A. sativa*	2
P80356.1	77	RecName: Full=Avenin-3; AltName: Full=Prolamin; Flags: Precursor	*A. sativa*	2
CBL51495.1	77	Avenin protein, partial	*A. sativa*	2
CCC80642.1	77	Avenin protein, partial	*A. canariensis*	2
CCC80654.1	77	Avenin protein, partial	*A. longiglumis*	2
CCC80666.1	77	Avenin protein, partial	*A. magna*	2
AGB56873.1	77	Gliadin-like avenin	*A. sativa*	2
AGB56869.1	77	Gliadin-like avenin	*A. sativa*	2
